# Striatal and Tegmental Neurons Code Critical Signals for Temporal-Difference Learning of State Value in Domestic Chicks

**DOI:** 10.3389/fnins.2016.00476

**Published:** 2016-11-08

**Authors:** Chentao Wen, Yukiko Ogura, Toshiya Matsushima

**Affiliations:** ^1^Graduate School of Life Science, Hokkaido UniversitySapporo, Japan; ^2^Department of Psychiatry, Graduate School of Medicine, Hokkaido UniversitySapporo, Japan; ^3^Japan Society for Promotion of SciencesTokyo, Japan; ^4^Department of Biology, Faculty of Science, Hokkaido UniversitySapporo, Japan

**Keywords:** reinforcement learning, temporal-difference learning, state value, striatum, tegmentum, domestic chicks, extinction learning

## Abstract

To ensure survival, animals must update the internal representations of their environment in a trial-and-error fashion. Psychological studies of associative learning and neurophysiological analyses of dopaminergic neurons have suggested that this updating process involves the temporal-difference (TD) method in the basal ganglia network. However, the way in which the component variables of the TD method are implemented at the neuronal level is unclear. To investigate the underlying neural mechanisms, we trained domestic chicks to associate color cues with food rewards. We recorded neuronal activities from the medial striatum or tegmentum in a freely behaving condition and examined how reward omission changed neuronal firing. To compare neuronal activities with the signals assumed in the TD method, we simulated the behavioral task in the form of a finite sequence composed of discrete steps of time. The three signals assumed in the simulated task were the prediction signal, the target signal for updating, and the TD-error signal. In both the medial striatum and tegmentum, the majority of recorded neurons were categorized into three types according to their fitness for three models, though these neurons tended to form a continuum spectrum without distinct differences in the firing rate. Specifically, two types of striatal neurons successfully mimicked the target signal and the prediction signal. A linear summation of these two types of striatum neurons was a good fit for the activity of one type of tegmental neurons mimicking the TD-error signal. The present study thus demonstrates that the striatum and tegmentum can convey the signals critically required for the TD method. Based on the theoretical and neurophysiological studies, together with tract-tracing data, we propose a novel model to explain how the convergence of signals represented in the striatum could lead to the computation of TD error in tegmental dopaminergic neurons.

## Introduction

To cope with the ever-changing environment, adaptive agents generate an internal representation of the value associated with their present state. Appropriate updating of state value is achieved through trial-and-error and model-free interactions with the environment. Based on the psychology of animal learning, a variety of reinforcement learning methods have been developed in the ongoing search for efficient updating processes. One such method is temporal-difference (TD) learning.

Historically, TD learning has stemmed from the finding of second-order conditioning (Pavlov, 1927), which the Rescorla-Wagner rule could hardly explain (Niv and Montague, 2009). TD learning makes such higher-order learning tractable, so that something that signals a predictor will also act as a predictor (Niv and Montague, 2009). According to the canonical formulation (Sutton and Barto, 1998), the value of the preceding state is updated by the TD error that is computed in the current state. Agents thus wait until the next time step to update the state value. This feature is particularly useful when agents are required to improve their strategy in a multi-step task, such as foraging animals or humans playing a board game. Variant algorithms have been developed to play games such as checkers (Samuel, 1959), backgammon (Tesauro, 1995), and Go (Silver et al., 2016), some of which have reached or even exceeded the level of expert human players.

These developments were accompanied by breakthroughs regarding the neuronal mechanisms of reinforcement learning in monkeys. In a multiple trial schedule, progress toward a forthcoming reward was represented in the striatum (Shidara et al., 1998) and the anterior cingulate cortex (Shidara and Richmond, 2002). Action-specific value representations have also been documented in the striatum (Samejima et al., 2005). Schultz *et al.* discovered that the dopaminergic neurons (DA neurons) in the ventral tegmental area (VTA) and the substantia nigra (SN) show a characteristic firing pattern that resembles TD-error signals (Montague et al., 1996; Schultz et al., 1997). In a later study, the signs of TD error signals have been shown to be inverted with respect to those of DA responses in the lateral habenula (Matsumoto and Hikosaka, 2007). Furthermore, the TD-error signals were also found in other brain regions; see (Schultz, 2015) for a comprehensive review. Despite the progress, it remains yet unclear how TD-error signals are computed. More specifically, questions remain regarding: (1) how the primary reinforcement and the current state value, i.e., the target signal, are represented; (2) how the preceding state value, i.e., the prediction signal, is represented; and (3) how these signals are merged to compute TD-error.

We sought to address these questions using domestic chicks as subjects. Within days of hatching, chicks can learn to peck conspicuous visual cues (i.e., a colored bead) to gain an associated reward (food and water) (Matsushima et al., 2003). In neuronal recordings from chicks performing the operant pecking task, both the medial striatum (MSt) (Yanagihara et al., 2001) and arcopallium (Arco, an associative area in the avian telencephalon, Aoki et al., 2003) have been found to code rewards and prediction of rewards. Particularly, some MSt neurons show a sequence of characteristic burst activities during the cue period, the post-operant delay period, and/or the reward period of the task (Izawa et al., 2005; Amita and Matsushima, 2014). Localized MSt lesions cause impulsive choices (Izawa et al., 2003) while Arco lesions cause cost-averse choices (Aoki et al., 2006), suggesting the involvement of these areas in foraging decision making. However, the functional roles of these multi-phase MSt activities remain unclear.

Several aspects of chick behavior are further puzzling. Generally, once acquired, pecks at rewarding cues are barely extinguished, although MSt lesions partially impair the process of extinction (Ichikawa et al., 2004). Even after the associated reward is omitted, chicks fail to stop pecking for several hours, as if the state value was not updated. However, when viewed by another behavioral measure, the chicks showed a quick change. Ichikawa et al. (2004) recorded how long chicks waited on the empty feeder, and found that they immediately started to decrease the waiting time from the first twenty trials after the onset of food omission. In the reward period, some aspects of state value are supposed to be quickly updated. Similarly, in another task in which chicks actively forage between two feeders placed at opposite ends of an I-shaped maze (Ogura and Matsushima, 2011; Ogura et al., 2015), chicks quickly changed their stay time when the profitability of the feeders changed (Xin et al., under review). Chicks behave as if the state value is flexibly updated, particularly in the final consumption phase when food reward is just to be gained.

In this study, we focused on the neuronal activities that occurred during the reward period when a predicted food was omitted. Specifically, we sought to distinguish the representations of the reward from those of the predicted reward. Preliminary recordings suggested that some MSt neurons could code the prediction even during the reward period, similar to GABAergic neurons in the VTA (Cohen et al., 2012). As the first step of our analysis, we constructed mathematical simulations of the critical signals of TD learning in the extinction task. As the second step, we classified recorded MSt neurons into three types according to changes in reward-period activity in the omission block. We then compared the simulated TD learning signals and the classified neurons. We analyzed neurons in the tegmentum around the substantia nigra (SN) and the formatio reticularis medialis mesencephali (FRM) in the same manner. As a third step, we examined the assumed connections from the MSt descending to the tegmentum via tract-tracing combined with immunostaining for thyroxine hydroxylase (TH, a marker of DA-ergic neurons). Based on these results, we propose a novel hypothetical process in which TD learning for foraging behavior is accomplished via interactions between the MSt and the midbrain DA system.

## Materials and methods

### Subjects and ethical note

Experiments were conducted according to the guidelines and approval of the Committee on Animal Experiments of Hokkaido University under the approval number 08-0500. The present protocol was initially approved on 22 January 2009, and thereafter on 14 March 2013 after a partial amendment. The guidelines are based on national regulations for animal welfare in Japan (Law for Humane Treatment and Management of Animals; after a partial amendment No.68, 2005). A total of 70 unsexed domestic chicks (*Gallus domesticus*, White Leghorn strain) were used in this experiment; 8 chicks for behavioral experiment (Figure S1), 45 chicks for neuronal recording in a freely behaving condition and 17 for neuroanatomical tract-tracing experiments. Fertilized eggs were purchased from a local supplier (Iwamura Poultry Ltd., Yubari, Japan) and incubated in the laboratory. We also used newly hatched male chicks from the same poultry supplier.

Training started on the day the chicks hatched (post-hatching day 0, or PH0). Prior to undergoing a surgical operation for chronic electrode implantation (PH7–8), pairs of chicks were housed in transparent plastic cages (30 × 17 × 13 cm) in a chamber lit by LED lamps on a 12-h light/12-h dark cycle, with the light phase starting at 09:00 am. After electrode implantation, the chicks were individually housed in the same chamber in transparent plastic cages (29 × 18 × 18 cm), so that chicks were mutually visible. Water was freely available from a drinking bottle, while food was strictly controlled. The restricted diet served to ensure that: (i) chicks actively fed during the behavioral tasks, and (ii) they increased in body weight (BW) gradually such that they reached 45 g or higher on PH7–8.

For the surgical operations, chicks were anesthetized via an intra-muscular injection of ketamine/xylazine cocktail (a 1:1 mixture of 10 mg/ml ketamine [Daiichi Sankyo Co., Ltd., Tokyo, Japan] and 2 mg/ml xylazine [Sigma-Aldrich Co., St. Louis, USA]) at a dose of approximately 0.1 ml per 10 g BW. Supplementary injections (0.1 ml) were given if necessary. When the brain was not sampled, chicks were euthanized via exposure to carbon dioxide.

### Apparatus

We used an operant box (30 × 28 × 38 cm, illuminated by LEDs and maintained at approximately 25–30°C) to train chicks and record single neuron activity in the freely behaving condition. The box was made of metal and electrically shielded to reduce noise. The subject chicks were monitored via a CCD camera on the ceiling of the enclosure, which enabled us to observe behaviors without being seen by the chicks. The ceiling was also equipped with a rotary slip ring, which enabled us to connect the implanted electrodes (tetrodes) to differential amplifiers located outside of the box.

**Figure 1:**
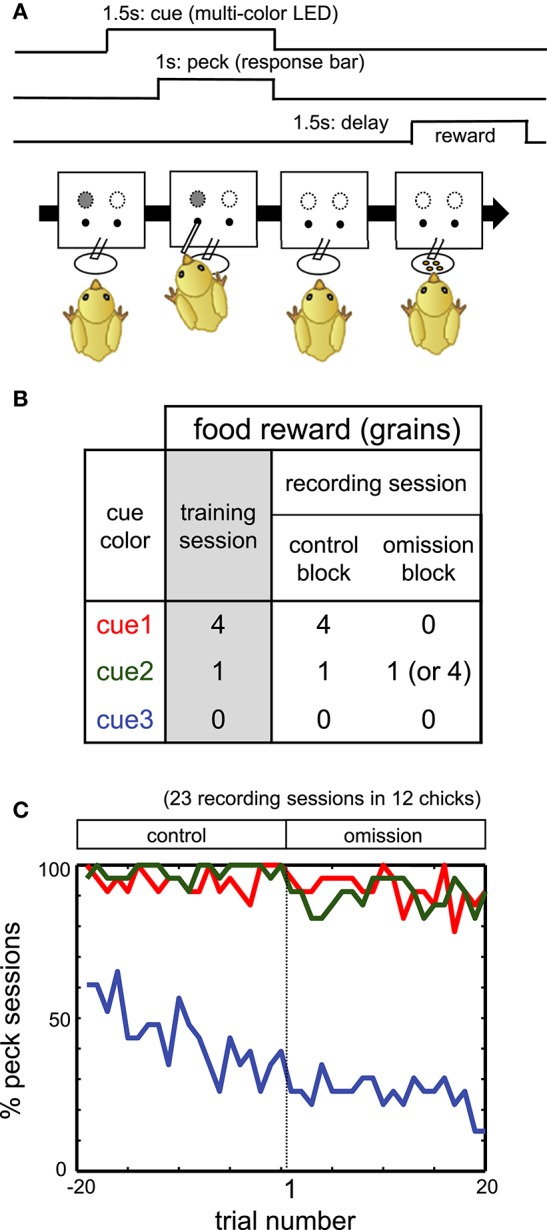
**(A)** Procedure of operant task reinforced by a delayed reward. **(B)** Color-food associations in training and recording sessions. **(C)** Percentage of the sessions in which chicks pecked. Red, green, and blue lines denote responses in cue1, cue2, and cue3 trials, respectively.

The front panel of the box was equipped with a pair of multi-color LEDs placed side by side (3.2 cm apart) for cue color presentation, a pair of holes through which the response bar protruded below the LEDs, and a feeder (a food-dispensing tube and dish) at the center of the panel; see Figure 1A. The LEDs, response bars, and feeder were driven by a micro-robot (RCX 1.0, Lego Co., Billund, Denmark) controlled via LabView (National Instruments Co., Austin, Texas, USA).

### Behavioral tasks

#### Habituation and pre-training

On PH0 and 1, pairs of chicks received one habituation session per day in the operant training apparatus. During each 20-min session, a multi-color LED (emitting red, green, or blue light) was circularly lit continuously, changing color in a fixed sequence every 40 s. Grains of millet seed were intermittently delivered in an unpredictable way that was not associated with the color of the lit LED.

On PH2, chicks were pre-trained to associate one of the LED colors (cue1) with a food reward (four grains of millet). In one trial, the LED was lit and the response bar was simultaneously protruded. The lit LED and the response bar were maintained until the chick pecked the bar. When the chick pecked the bar, the LED was immediately turned off and the bar was retracted, and the food was delivered without a delay. We conducted two pre-training sessions (20 min each) on PH2. In the first session, chicks were trained in pairs. In the second session, chicks were individually trained. After these two sessions, individual chicks were tested for their responses to the simultaneously presented LED and bar in 20 consecutive trials. Those chicks that pecked the bar in 15 or more trials were subsequently trained in the color-food association task.

#### Color-food association training

On PH3–5, chicks were trained to associate the LED colors with the following food rewards: four grains (cue1), one grain (cue2), and no food (cue3). See Figures 1A,B for the schedule of trials and the color-reward associations. The assignment of the LED colors (red, green, or blue) to cue1–3 was randomized among individuals. In each trial, one of the cue LEDs was lit, and its onset was defined as *t* = 0 s. After 0.5 s (*t* = 0.5 s), a response bar protruded for the chick to peck. The chick had 1 s to peck the bar. Irrespective of whether the chick pecked, the LED turned off and the bar was retracted at *t* = 1.5 s. After a delay period of 1.5–2.0 s (*t* = 3.0–3.5 s), chicks received the associated food. If chicks did not peck the bar, food was not delivered. If chicks responded incorrectly (no peck at cue1 and cue2, or peck at cue3), correction trials were repeated, with up to five additional trials.

Individual chicks received two training sessions per day. One session comprised 60 trials, excluding correction trials: 20 trials for each cue1, cue2, and cue3, with a pseudo-random order of presentation. Inter-trial intervals ranged from 12 to 20 s. On PH6 or afterward, i.e., after 3 days of training, a final test was conducted. The test procedure was identical to the training sessions on PH3–5, except that no correction trials were given. If chicks pecked the bar in ≥ 17/20 trials for both cue1 and cue2, and ≥ 10/20 trials for cue3, they proceeded to the electrophysiological experiment. If chicks failed to meet these criteria on PH6, they were repeatedly trained and tested up to PH8. Those chicks that met the criteria on PH8 also proceeded to the electrophysiological experiment.

#### Behavioral task during electrophysiological recording

To investigate how the neuronal correlates of food reward are updated, we recorded extracellular single unit activities from freely behaving chicks performing a food omission task (Figure 1B). The recording session comprised an initial control block followed by an omission block. Cue1 was associated with four grains of food in the control block. In the omission block, for cue 1, food was omitted (in 42 chicks) or delivered after a longer delay period of 3.5 s (in three chicks). After electrophysiological recording in the omission block, chicks were re-trained in the same condition as the control block (termed the reacquisition block) before the next recording session. If not stated otherwise, the association for cue2 and cue3 did not change.

### Recording of single unit activity

#### Chronic implantation of tetrode

We recorded neuronal activity using tetrodes, which were hand-made by twisting 4 formvar-insulated nichrome wires (bare diameter: 18 μm; coated diameter: 25 μm; A-M System Co., Sequim, Washington, USA). The tip of each tetrode was gold-plated and its resistance was reduced to 100–300 kΩ when measured at 1 kHz in a saline solution. We used a metal electrode impedance tester (Model IMP-2, Bak Electronics, Inc., Umatilla, Florida, USA) for the impedance measurements. The plated tetrodes were inserted in thin stainless steel tubes, implanted into the brain tissue, and connected to a micro-driver.

On PH7 or 8, chicks were anesthetized as described above. The anesthetized chicks were fixed on a rat stereotaxic apparatus (type SR-5N, Narishige, Co. Tokyo, Japan) modified such that it was possible to secure the beak of a chick. Using micromanipulators (type SM-15M), a tetrode was inserted into either the medial striatum or midbrain tegmentum. The coordinates of the tetrode tips are shown in Table S1. After the tetrode reached the coordinates, the micro-driver was chronically fixed to the skull surface with dental cement, allowing us to gradually insert the tetrode.

#### Amplifiers for extracellular recording

Recording started on the day after tetrode implantation. Neuronal signals were buffered by a head-amplifier (FET input operational amplifier, TA75074F, Toshiba, Tokyo, Japan) and then amplified by an AC-coupled differential amplifier. The cut-off frequency was set at 0.3 kHz, amplification × 2000, and the band-pass filter was set at 0.5–1.5 kHz (18 dB per octave). Signals were A/D-converted at a sampling rate of 16.6–25.0 kHz (Micro1401, CED Co., Cambridge, UK) and stored in a PC. For technical notes on separations among single units, see Figures S4-1–S4-3.

#### Histological examination of recording sites

After the recording experiment, chicks were given an overdose of ketamine/xylazine cocktail (0.6–0.7 ml of a 1:1 mixture) and transcardially perfused with a fixative (4% paraformaldehyde in 0.1 M PB; PFA). The entire brain was dissected out and post-fixed for up to 1 week in the same fixative at 4°C. The brain tissue was then trimmed, embedded in egg yolk, and fixed for an additional 3 days. The tissue was subsequently cut into a complete series of 50-μm-thick frontal sections using a vibrating microtome (DTK-3000, Dosaka Co., Kyoto, Japan). Sections were mounted on glass slides coated with APS (amino silane), stained with cresyl violet, cover-slipped, and examined using a microscope and a drawing tube. The recording sites were estimated based on the complete reconstruction of tetrode tracks and record of tetrode advancement. The coordinates conformed to the chick brain atlas (Kuenzel and Masson, 1988), and neural nuclei terminology conformed to the nomenclature reform (Reiner et al., 2004).

### Tract tracing by BDA and DiI

To reveal the efferent terminals from the MSt, we used biotinylated dextran amine (BDA, 0.1 μl per injection, 10% in distilled water, 10 kDa; D22910, Molecular Probes®, Thermo Fisher Scientific Inc., USA) as an anterograde tracer. To reveal the MSt neurons projecting to the SN, we used 1,1′-dioctadecyl-3,3,3′3′-tetramethylindocarbocyanine perchlorate (DiI, 30 nl per injection, 7% solution in N,N-Dimethylformamide) as a retrograde tracer. We used a micro injection instrument (Nanoject II, Drummond Scientific Co., Broomall, Pennsylvania, USA) to inject the tracer into chicks aged approximately PH9. The injection was performed under ketamine/xylazine anesthesia, as described above. Either 7 days (BDA) or 11 days (DiI) after the operation, chicks were transcardially perfused with 4% PFA. Brains were dissected out and post-fixed in the same fixative at 4°C overnight (BDA) or for ≥ 3 days (DiI).

#### Histochemistry for visualizing anterograde labeling with BDA

After 1 day of cryo-protection in PBS with 20% sucrose, the brains were frozen and stored at -30°C until sectioning. We used a sliding microtome with a freezing stage (TU-213, Yamato Kohki Industrial Co. Ltd., Saitama, Japan) to cut the brains into sagittal sections for single or double histochemical labeling.

For single labeling to visualize BDA, 60-μm-thick sections were cut and incubated in avidin-biotinylated horseradish peroxidase complex reagent (PK-6100, Vectastain® Elite ABC Kit, Vector Laboratories Co., USA) and DAB (SK-4100, DAB Peroxidase Substrate Kit, Vector Laboratories) as a chromogen. Sections were mounted on APS coated glass slides (S8441, Matsunami Glass Ind. LTD., Osaka, Japan), cover-slipped in Permount™ mounting medium (SP15-500, Thermo Fisher Scientific Inc., USA), and stored at room temperature.

For double labeling, 24-μm-thick sections were initially soaked in Alexa Fluor®488 streptavidin conjugate at room temperature for 1 h to visualize BDA (S32354, Molecular Probes®; dilution by 1:400). The sections were then processed with a primary antibody; rabbit anti-TH (1:1000, 4°C, overnight; AB152, Chemicon®, EMD Millipore Co., USA) or rabbit anti-GAD65 (1:1000, 4°C, 3 days; bs-0400R, Bioss Inc., USA). As the secondary antibody, we used goat anti-rabbit IgG - Alexa Fluor® 568 conjugate (1:400, A11011, Molecular Probes®) at room temperature for 1 h. Sections were then mounted on APS coated glass slides and cover-slipped in Prolong® Diamond antifade mountant with DAPI (P36962, Thermo Fisher Scientific Inc.) and stored at room temperature.

#### Procedure for retrograde labeling with DiI

The fixated brains were embedded into yolk, post-fixated in 4% PFA for an additional ≥ 3 days, and cut into 50-μm-thick sections using a vibrating microtome (DTK-1000). Sections were collected in PBS, mounted onto APS coated glass slides, and cover-slipped in PBS. The cover glass was sealed with a transparent nail polish to prevent drying.

#### Microscopic observations

At low magnification, stained sections were photographed using a bright-field light microscope (Olympus BH-2) and fluorescence microscopes (Leica MZ16F and EVOS® FL Imaging System). We used a confocal microscope (Zeiss LSM 510) to examine the connectivity between BDA-positive terminals and tegmentum neurons at a high magnification. Scanned images were examined using Zeiss LSM 5 Image. Images of interest were edited using a free graphics editor GIMP2.8 (GNU Image Manipulation Program; URL: https://www.gimp.org/).

## Results

### Simulation of neuronal representations of temporal-difference learning

**Figure 2:**
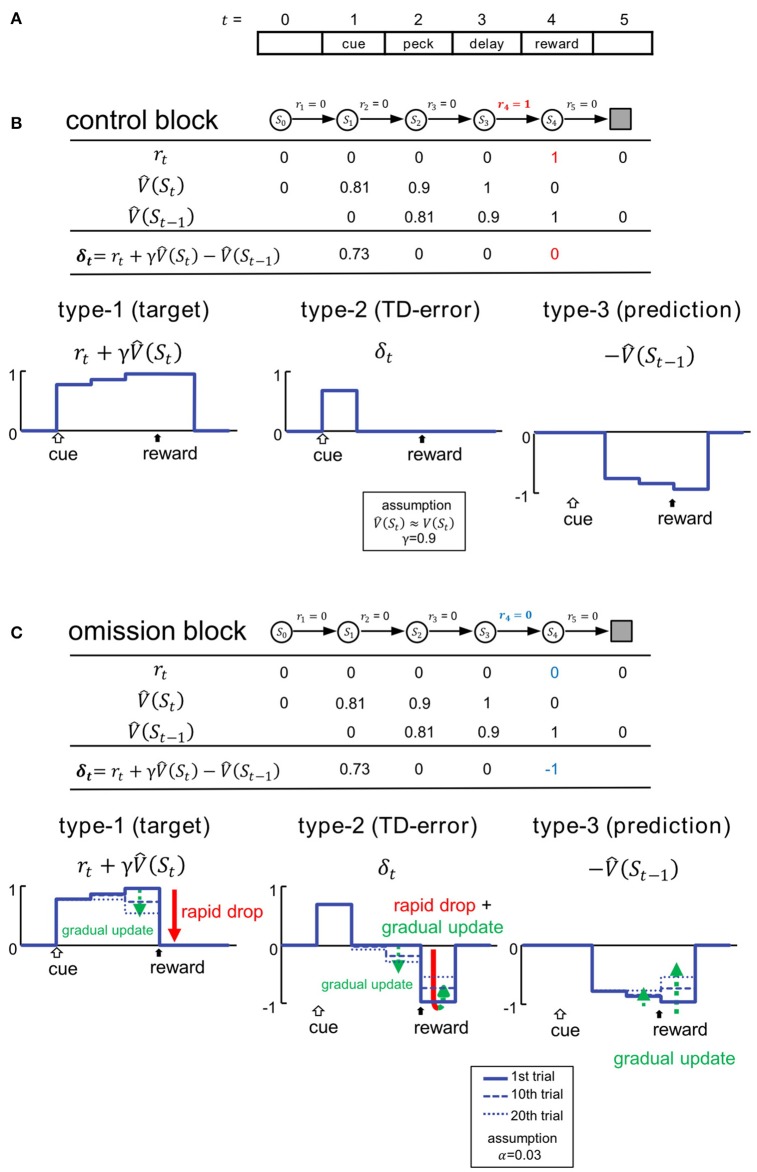
**Variables of TD learning were simulated according to the task**. **(A)** Trials were mimicked as finite sequences composed of 6 discrete states. **(B)** Control block. Signals are shown in the table and also schematically illustrated below. *S*_*t*_ and *r*_*t*_ denote state and reward, and $\hat{V}\left(S_{t}\right)$ represents the estimated state value at *t*. δ_*t*_ represents the temporal difference error (TD error). Temporal discounting of reward was not assumed, thus γ = 0.9. **(C)** Omission block. Signals in the first trial of the omission block are shown in the table, and their updating processes are illustrated below. Signals at the 1st, 10th, and 20th trial are shown. We adopted TD(0) method, and assumed the learning rate to be α = 0.03. For an additional simulations with α = 0.06, see Figure S3.

To simulate critical signals involved in TD learning, we assumed a discrete step-time procedure that mimicked the behavioral task (Figure 2A). We adopted an algorithm that followed the standard formulation of the one-step TD method (TD(0) method) (Sutton and Barto, 1998). In this simulation, a trial is a finite sequence composed of states *S*_0_, *S*_1_, *S*_2_, *S*_3_, *S*_4_ and *S*_*terminal*_, corresponding to a pre-trial period (*t* = 0), cue period (1), peck-operant period (2), delay period (3), and reward period (4), respectively, followed by the terminal. At the transition to each state S_*t*_, reward is received (*r*_*t*_ = 1) or not (*r*_*t*_ = 0). In the control block, as the reward was delivered in the reward period, we set *r*_4_ = 1 and *r*_*t*_ = 0 when *t* ≠ 4, with an arbitrary unit (Figure 2B).

Generally, the state value *V*(*S*_*t*_) is given by the expected sum of the discounted future rewards after *S*_*t*_, such as:

(1) $$\begin{array}{l} V\left(S_{t}\right)=E\left[r_{t+1}+\gamma r_{t+2}+\gamma^{2}r_{t+3}+\ldots \;\right] \end{array}$$

For simplicity, we hypothesize the temporal discounting γ = 0.9. *V*(*S*_*t*_) is hidden, and subjects must learn to estimate it through experience. In Figure 2B, $\hat{V}\left(S_{t}\right)$ denotes the subjective estimate of *V*(*S*_*t*_). We assumed that subjects had been fully trained, so $\hat{V}\left(S_{t}\right)=V\left(S_{t}\right)$ in the control block. We therefore assume *V*(*S*_*t*_) (and thus $\hat{V}\left(S_{t}\right)$) for *t* = 1 to 3 (Figure 2B). In the first trial of the omission block, $\hat{V}\left(S_{t}\right)$ is equal to that in the control block (Figure 2C), even though *r*_4_ turns = 0 (Figure 2C). In subsequent trials, $\hat{V}\left(S_{t}\right)$ is gradually updated according to the TD(0) method, so that:

(2) $$\begin{array}{l} \hat{V}\left(S_{t-1}\right)\leftarrow \hat{V}\left(S_{t-1}\right)+\alpha \delta_{t} \end{array}$$

where α ∈ [0, 1] is the learning rate. In this scheme, α is set as 0.03. The TD error δ_*t*_ is given by:

(3) $$\begin{array}{l} {\;\delta }_{t}=r_{t}+\gamma \hat{V}\left(S_{t}\right)-\hat{V}\left(S_{t-1}\right) \end{array}$$

We assume that neurons in the medial striatum and tegmentum represent the critical signals in the formula (3) (Figures 2B,C). Thus, in addition to the target of TD learning $r\left(t\right)+\;\gamma \hat{V}\left(S_{t}\right)$, predicted rewards are also represented in terms of delayed and inhibitory activity in the form of $-\hat{V}\left(S_{t-1}\right)$. In other words, reward prediction signal can also appear in the reward period (i.e., $-\hat{V}\left(S_{3}\right)$.), and is represented as inhibition, or suppressed neuronal activity. As formula (3) indicated, the simple summation of these two signals will yield the TD error signal δ_*t*_. In the following, these signals are referred to as type-1, -3, and -2. We compared these signals: $r_{t}+\gamma \hat{V}\left(S_{t}\right)$, $-\hat{V}\left(S_{t-1}\right)$, and δ_*t*_, with activities recorded from neurons in the medial striatum (**Figures 4, 5**) and tegmentum (**Figures 7, 8**). We paid particular attention to the characteristic temporal patterns of neuronal activities in each trial, and their changes in the omission block.

In the control block (Figure 2B), the type-1 signal is turned on at the cue period, and shows sustained activity through the peck/delay/reward periods. The activity of the type-2 signal is similar, but the delayed inhibition is due to the type-3 signal which cancels out the peck/delay/reward period activities, while the initial transient activity still remains as the TD error δ_*t*_. In the omission block (Figure 2C), as the reward signal *r*_4_ turns = 0, both type-1 and -2 signals will show a rapid drop in activity during the reward period. Type-1 activity will drop to the level of baseline, whereas type-2 activity will drop below baseline. Conversely, activity in type-3 signals will remain unchanged in the first trial of the omission block. In subsequent trials, the activities of all three types of signals will be gradually updated. Specifically, the state value in the reward period will be updated as follows:

$$\begin{array}{l} \hat{V}\left(S_{3}\right)\leftarrow \hat{V}\left(S_{3}\right)+\;\alpha \delta_{4}\;\left(\alpha =\;0.03\right)\;\left(2^{\prime }\right) \end{array}$$

Based on the updating rule (2′), we simulated 3 dynamic values: *r*_4_, δ_4_, and $\hat{V}\left(S_{3}\right)$ in the omission block. We constructed 3 corresponding statistical models for classifying real neurons: the Actual Reward (AR) model for type-1, Prediction Error (PE) model for type-2, and Reward Prediction (RP) model for type-3. See the Appendix in Supplementary Material for details regarding the statistical models. Please note α is a free parameter which can be estimated based on the activities in each neuron. *Post hoc* analyses revealed that the value α = 0.03 is reasonable (see Figure S11). Note also that γ was not critical in these models, which tried to simulate the reward period activities.

### MSt neuron activities

#### Habitual pecking responses in the omission block

Throughout the initial part of the omission block, for at least 20 trials, the chicks pecked the response bar for both cue1 and cue2. In Figure 1C, the percentage of sessions in which the subject chick pecked (y-axis) was plotted against the trial number (x-axis; −19 to 0 in the control block, 1 to 20 in the subsequent omission block). The figure shows data obtained from 12 chicks in 23 recording sessions in which neuronal activities were successfully recorded. In the omission block, for both cue1 (red) and cue2 (green) trials, chicks pecked the response bar in ≈ 90%, even though food was omitted for cue1 but not for cue2. In contrast, the pecking response in the cue3 trials (non-rewarding trials) monotonically deceased through both blocks (blue). We therefore assumed that the pecking response was habituated at least for the initial 20 trials in which neuronal activities were recorded.

On the other hand, as has been reported previously (Ichikawa et al., 2004), reward omission gave rise to an immediate change in the chick behavior during the reward period. A supplementary experiment revealed that the waiting time (or “giving up time”) at the empty feeder started to decrease in the first 20 trials of omission, even though the chick pecked the response bar in the cue1 trials (Figure S1). Evident decrease occurred in the cue1 trials, while it was less clear in the cue3 trials. It is therefore appropriate to assume that chicks update the reward expectations during the reward period from the early phase of the omission block. In the following, if not stated otherwise, we analyzed activity during the initial 20 trials of the omission block.

#### General properties of MSt neurons

**Figure 3:**
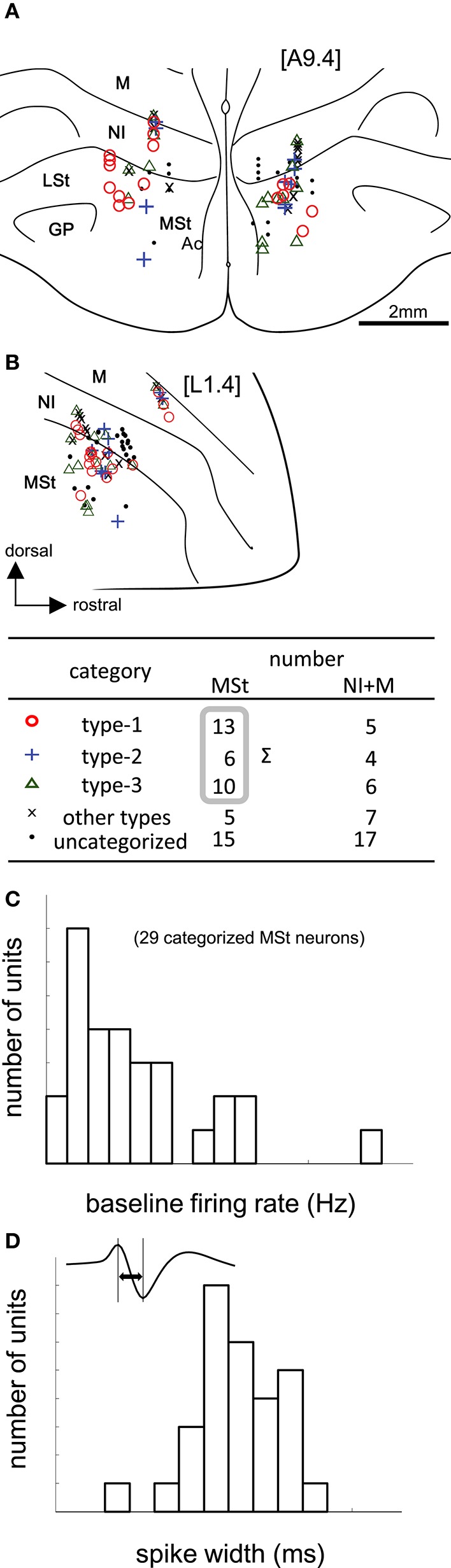
**Task-related neurons in the medial striatum (MSt) and surrounding regions**. **(A,B)** Histological reconstruction of recording sites on frontal **(A)** and sagittal **(B)** planes. Anterior-posterior level [A9.4] and laterality level [L1.4] follow the atlas by Kuenzel and Masson (Kuenzel and Masson, 1988); see Appendix Supplementary Material for abbreviations. Symbols denote different neuron types (inlet table). Neurons were categorized as type-1, -2, -3, or other, according to the reward period activities of the cue1 trials. **(C)** Baseline firing rates (pre-trial period) of 29 MSt categorized neurons in a histogram. **(D)** Spike width, as measured by the peak-to-peak duration (inlet figure), in a histogram.

**Figure 4:**
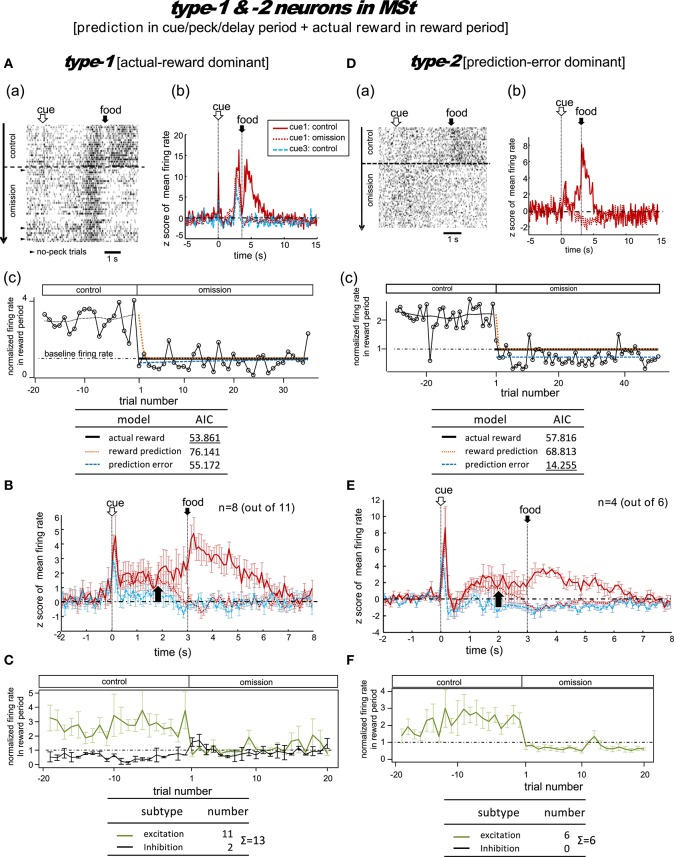
**MSt neurons representing reward prediction in the cue/peck/delay periods, and actual reward in the reward period**. These neurons were categorized into two types; type-1 (actual-reward dominant; **A–C**) and type-2 (prediction-error dominant; **D–F**). Panel **(A)** shows a representative example. In **A(a)**, activities in cue1 (rewarding) trials are shown as a rastergram. Arrowheads indicate the few no-peck trials in which chicks did not peck the response bar. In **A(b)**, the averaged firing rate (z-score) in the cue1 trials is compared between the control block (4 grains: red line) and the omission block (0 grain: dashed red line). Activity in the non-rewarding cue3 trials in the control block is superimposed (dashed blue line). In **A(c)**, the normalized firing rate in the reward period (open circles connected with lines) is plotted against the trial number, with the number = 1 denoting the first trial of the omission block. The dashed line superimposed in the control block represents the smoothed activity. We tested the fit of the firing rate in the omission block to three models: actual reward (thick dark line), reward prediction (orange dotted line), and prediction error (blue dashed line). The table below shows the AIC value of each fit curve; see the text for details regarding the models. **(B)** Shows population data; mean firing rate (z score) of type-1 neurons (*n* = 8, excitation type; mean and s.e.m.) in cue1 (control; red line), cue1 (omission; dashed red line), and cue3 (control; dashed blue line) trials. The upward arrow indicates the divergence point between the control and omission block for cue1. **(C)** Normalized firing rates of type-1 neurons in the reward period. Excitation (green line) and inhibition type (black line) neurons are shown separately. A representative example **(D)** and population data **(E–F)** for type-2 neurons, which had significant inhibition in the reward period, thus fitting the prediction error model well. Panels **(D–F)** follow the same conventions as **(A–C)**.

**Figure 5:**
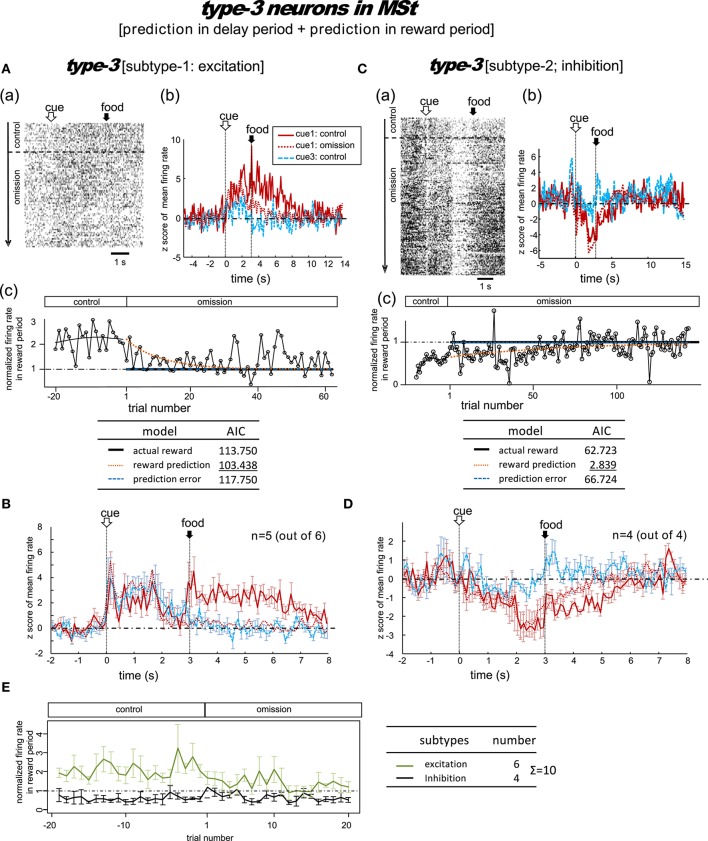
**MSt neurons with representations of reward prediction in the delay period and the reward period (type-3, reward-prediction dominant)**. Activity in the cue1 trials of the omission block fit best to the reward prediction model. **(A,B)** Excitation subtype. **(C,D)** Inhibition subtype. **(E)** Changes across the trial number. In contrast to the type-1/2 neurons in Figure 4, the delay-period activity did not diverge between the control and omission blocks.

We recorded 88 neurons in 68 recording sessions from 30 chicks. Histological examination revealed that these neurons were located in the MSt, nidopallium intermedium (NI), and mesopallium (M) (Figures 3A,B). In the present study, we focused on MSt neurons, and NI and M neurons were disregarded. In 34 out of the 49 neurons in the MSt, we recorded activity for a sufficiently long duration, enabling us to classify the neurons by type. Of 29 neurons, we found 13 to be type-1, 6 to be type-2, and 10 to be type-3; 5 neurons failed to match any model, and were assigned to an “other” category. A one-way unbalanced ANOVA revealed a difference in the laterality of the recording sites among the three types (*F* = 4.34, df = 2, *p* = 0.0236). Type-1 neurons were located more laterally than type-3 neurons according to a *post hoc* Tukey test (*p* = 0.0228; type-1: 1.92 ± 0.58 mm; type-3: 1.28 ± 0.55 mm, mean ± SD). In the anterior-posterior level, we found no significant difference among the three types (ANOVA: *F* = 1.08, *p* = 0.355). In terms of baseline firing rate and spike width, an ANOVA revealed no significant difference among the three types (Figure 3C, firing rate: *F* = 0.28, *p* = 0.755), (Figure 3D, spike width: *F* = 1.02, *p* = 0.375). In the following sections (Figures 4 and 5), we show the neuronal activities in terms of (1) z scores of the averaged firing rate in each block, and (2) temporal changes in the normalized firing rate in the reward period, plotted across trials.

#### Type-1 neurons

Figure 4A shows a representative example. In the control block, after a brief period of transient activity after cue1 onset, tonic responses appeared in the delay period and the reward period. In the omission block, the reward-period response disappeared immediately from the first trial. The cue1 activity in the reward period was identical to that of cue3, in which the food reward was also absent. We thus assumed that this neuron coded the actual reward in the reward period. The actual reward model gave rise to the smallest AIC (inlet table), thus confirming our assumption as type-1. In contrast, the transient activity after cue onset and the tonic activity in the delay period remained in the omission block, although we observed a slight decrease in amplitude. This feature supports the idea that the neuron also coded reward prediction prior to food delivery. However, this neuron also fired, although weakly, in the delay period of the cue3 trials in which no reward followed. Despite this, the simulated target signal $r_{t}+\gamma \hat{V}\left(S_{t}\right)$ (Figure 2) was a good fit for this neuron.

We categorized a total of 13 MSt neurons as type-1 neurons based on their reward-period activities; 11 neurons showed excitation and two showed inhibition during the reward period. Of these 11 excitation subtype neurons, we averaged the activities of eight neurons for their firing rate (z scores, Figure 4B). The other three neurons were not included as the recording time was less than 20 trials in the omission block. The rapid drop in reward-period activity was also reproduced (Figure 4C). The actual-reward activity was preceded by a reward-prediction signal during the delay period. Note that the delay-period activity declined in the omission block, as indicated by the upward arrow. In contrast, no decline was observed in the cue/peck periods. In an example neuron, shown in Figure S5, a normalized firing rate in the delay period (1.5–3.0 s) gradually declined during the omission block, suggesting a gradual change in the reward prediction, in concert with the simulated $r_{t}+\gamma \hat{V}\left(S_{t}\right)$ signal (Figure 2C). Two neurons showed inhibition during the reward period. These neurons showed complex firing patterns that were markedly different from those of the other neurons (see Figure S6).

#### Type-2 neurons

Figure 4D shows a representative example. In the control block, similar to the type-1 example, transient activity upon cue1 onset was followed by tonic responses in the delay and reward periods. However, in the omission block, the reward-period activity changed its sign immediately from excitatory to inhibitory. Cue3 trials were not conducted in this recording. We thus assumed that this neuron coded the negative prediction error in the reward period. The prediction error model gave rise to the smallest AIC (inlet table), thus confirming the status of type-2 neuron. The sign of the tonic activity in the delay period was also inverted in the omission block. However, the transient activity after the cue onset remained. This feature indicates that the neuron also coded reward prediction in the cue period. The prediction signal in the cue period and the negative prediction error signal in the reward period fit the simulated TD-error signal δ_*t*_. However, the excitatory response to the predicted-reward conflicted with the simulated δ_*t*_ (Figure 2B).

We categorized a total of six MSt neurons as type-2 based on their reward-period activities. All six neurons showed excitation during the reward period. Of these six neurons, we averaged the firing rate of four neurons (z scores, Figure 4E); the other two neurons were not included due to insufficient recording time. The rapid drop and inverted sign of the reward-period activity were reproduced (Figure 4F). Similar to the type-1 neurons, we observed the reward-prediction signal during the delay period, and found that this signal declined in the omission block (upward arrow). In contrast, cue/peck period activity did not decline. The rapid drop, inverted-sign, and declined delay-period activity fit the simulated δ_*t*_ (Figure 2C). However, both (1) the excitatory reward-period activity in the cue1 trials in the control block and (2) the inhibitory reward-period activity in the cue3 trials in the control block conflicted with the simulated δ_*t*_ (Figure 2B), which showed no response in the reward period for these two cases.

#### Type-3 neurons

A total of 10 MSt neurons were categorized as type-3 neurons based on their reward-period activities: six neurons showed excitation and the other four neurons showed inhibition during the reward period.

##### Excitation subtype

Figure 5A shows a representative example. In the control block, tonic responses appeared in the peck, delay, and reward periods. In the omission block, the reward-period response gradually disappeared. The cue1 activity was still higher than the cue3 activity in the delay and reward periods, but it was lower than the cue1 activity in the control block. This neuron was thus assumed to code for reward prediction both prior to and after food delivery. The reward prediction model gave rise to the smallest AIC (inlet table), thus confirming the status of type-3 neuron. In cue3 trials with no reward, this neuron also fired in the delay period, but much more weakly. The simulated prediction signal $\hat{V}\left(S_{t-1}\right)$ (Figure 2) fit this neuron well.

Of the six excitation subtype neurons that we found, we averaged the firing rate of five neurons (z scores, Figure 5B); the other one neuron was discarded due to insufficient recording time. The gradual decrease in reward-period activity was reproduced (Figure 5E, green). The reward-prediction activity was preceded by the reward-prediction signal during the cue/peck periods, which remained in the omission block. However, the delay-period response was absent. This conflicts with the simulated $\hat{V}\left(S_{t-1}\right)$ signal (Figure 2).

##### Inhibition subtype

Figure 5C shows a representative example. In the control block, a brief inhibitory transient activity in the cue period was followed by inhibitory tonic responses in the delay and reward periods. In the omission block, the reward-period response remained and gradually disappeared. Paralleling the slow change in neuronal activity, the no-peck trials became gradually more frequent (see Figure S2). In contrast, the cue3 activity in the reward period was near baseline levels. We thus expected this neuron to code for reward prediction in the reward period. The reward prediction model gave rise to the smallest AIC (inlet table), thus confirming the status of type-3 neuron. In contrast, the transient activity after cue onset and tonic activity in the delay period remained in the omission block, with nearly the same amplitude. This finding supports the idea that the neuron also coded the reward prediction prior to the food delivery. This neuron showed nearly no response in cue3 trials in which no reward followed the cue. The simulated prediction signal $-\hat{V}\left(S_{t-1}\right)$ (Figure 2) fit this neuron well.

As a special case, we tested the response of this neuron when the number of grains of food increased from 1 to 4 in cue2 trials. The amplitude of the inhibitory response in the reward period gradually increased (Figure S7). This result supports our expectation that reward-period activity in this neuron codes reward prediction.

We then averaged the firing rate of the four inhibition subtype neurons (z scores, Figure 5D). We found that the gradual change in reward-period activity in the omission block was reproduced (Figure 5E, black). The reward-prediction activity was preceded by a reward-prediction signal during the delay period, which remained in the omission block. The weak cue/peck period responses also remained in the omission block. All of these features are consistent with the simulated $-\hat{V}\left(S_{t-1}\right)$ signal (Figure 2).

### Tegmentum neuron activities

#### General properties of tegmentum neurons

**Figure 6:**
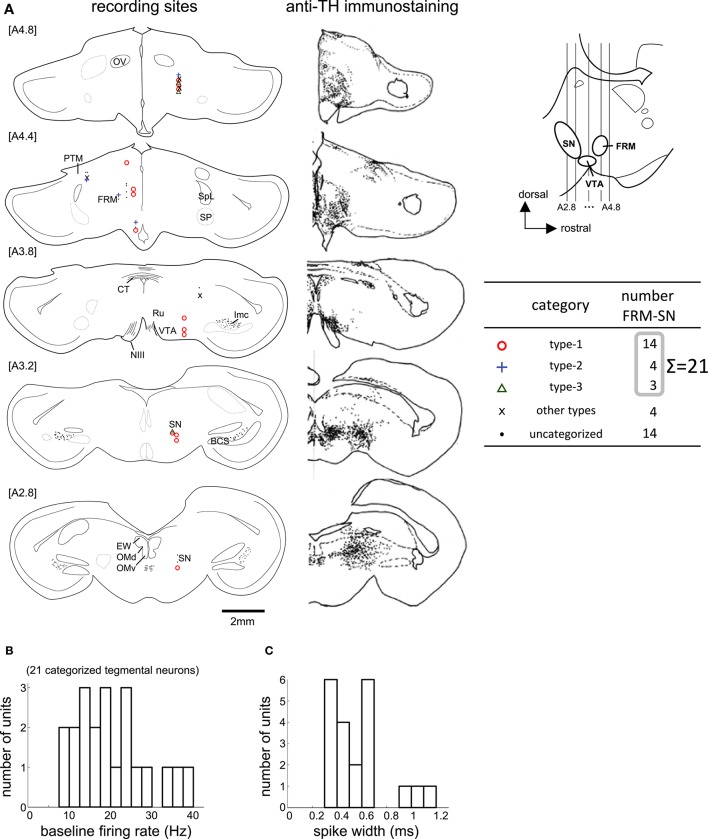
**Task-related neurons in the midbrain tegmentum. (A)** Histological reconstruction of recording sites on frontal planes, corresponding to the anti-TH immunostaining on the right; see Appendix in Supplementary Material for abbreviations. The anterior-posterior level of sections (A2.8 to A4.8) is shown in the inlet. **(B,C)** Baseline firing rate and spike width histograms of the 21 categorized tegmentum neurons.

**Figure 7:**
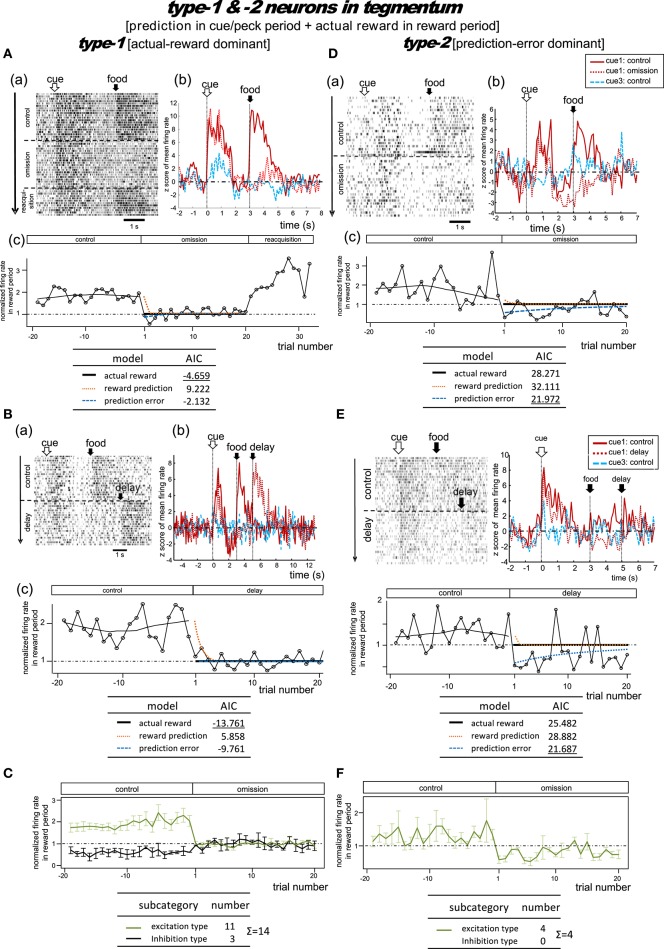
**Tegmental neurons representing reward prediction in the cue/peck period, and actual reward in the reward period**. These neurons were categorized into two types; type-1 (actual-reward dominant) and type-2 (prediction-error dominant). Two example neurons are shown for each type. Panels **(A)** and **(B)** show two type-1 neurons tested in the omission **(A)** or in the condition with delayed delivery of reward **(B)**. Similarly, **(D)** and **(E)** show two type-2 neurons tested in the omission **(D)** or delayed reward condition **(E)**. **(C,F)** Normalized firing rate plotted against the trial number for type-1 **(C)** and type-2 **(F)** neurons (population data). Mean ± SEM (error bars).

**Figure 8:**
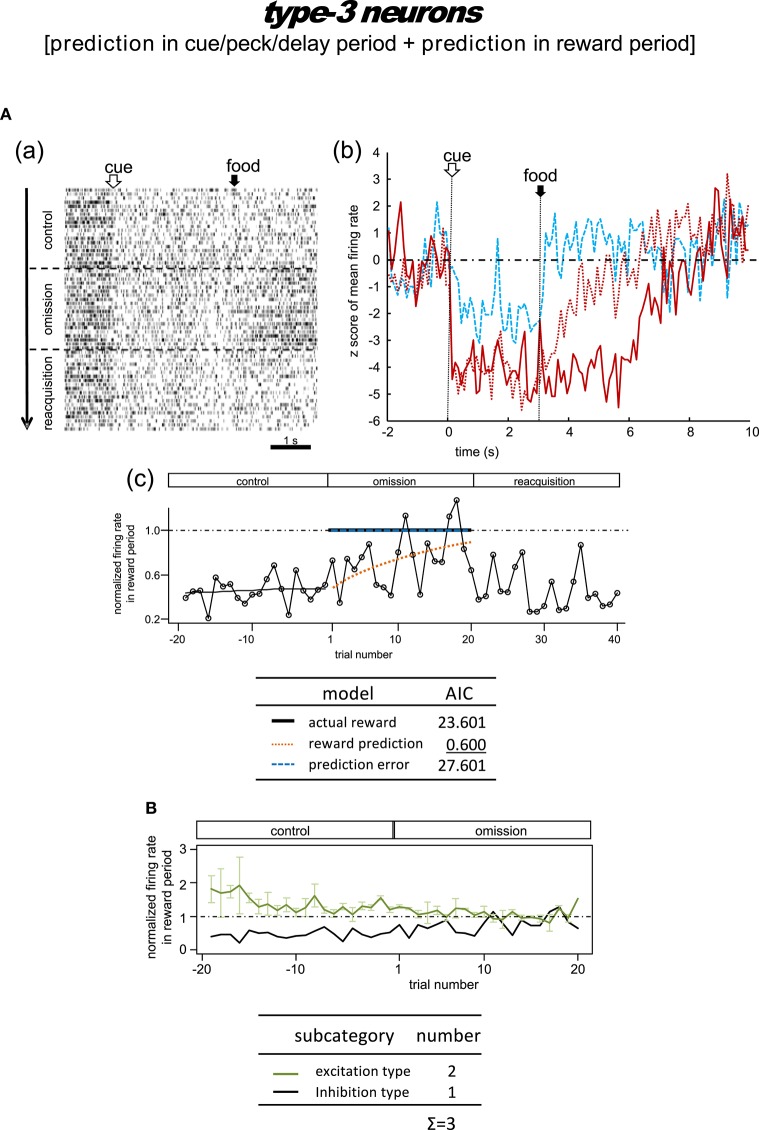
**Tegmental neurons representing reward prediction in the cue/peck/delay period and the reward period (type-3, reward-prediction dominant)**. The omission block was followed by another rewarding block (reacquisition) in the example shown in **(A)**. Note the gradual decline in reward-period inhibition in the omission block in **A(b,c)**. **(B)** Normalized firing rate plotted against the trial number for type-3 neurons (population data). Mean ± SEM (error bars).

We recorded 39 neurons in 36 recording sessions from 15 chicks. Histological examination revealed that these neurons were located in the SN, FRM, and other regions rich in DA neurons (Figure 6A). In 25 out of the 39 neurons, activity was recorded for a sufficiently long period, enabling us to classify the neuronal type. Of the 25, 14 were type-1, 4 were type-2, and 3 were type-3. Four neurons failed to match any model, and were assigned to an “other” category. The type-1 neurons were widely distributed in all five anterior-posterior levels. The type-2 neurons were found in the [A4.8] and [A4.4] levels. The type3 neurons were sparsely distributed in the [A4.8] and [A3.2] levels. In terms of baseline firing rate and spike width, an ANOVA revealed no significant differences among the three types (Figure 6B, firing rate: *F* = 0.31, *p* = 0.735), (Figure 6C, spike width: *F* = 0.63, *p* = 0.543). The tegmentum neurons were recorded in the omission condition or in the delay condition (i.e., the delay period increased by 2 s). In the following sections (Figures 7, 8), we describe the neuronal activities in these 2 conditions separately.

#### Type-1 neurons

##### Omission condition

Figure 7A shows a representative example. In the control block, tonic responses appeared in the cue/peck period and the reward period. In the omission block, the reward-period activity disappeared immediately from the first trial onwards. In the following reacquisition block, the reward-period activities reappeared within the first trial. We found no cue3 activity in the reward period. We thus expected this neuron to code for the actual reward in the reward period. The actual reward model gave rise to the smallest AIC (inlet table), thus confirming the status of type-1 neuron. Conversely, the tonic cue/peck period activity remained in the omission block. The cue3 activity in the cue/peck period was weaker than that for cue1. These features support the idea that the neuron also coded the reward prediction prior to the food delivery. However, this neuron lacked a response in the delay period, which conflicts with the simulated target signal $r_{t}+\gamma \hat{V}\left(S_{t}\right)$ (Figure 2).

##### Delay condition

Figure 7B shows a representative example. In the control block, two tonic responses appeared in the cue/peck period and the reward period. In the omission (delay) block, the reward-period response disappeared immediately from the first trial onwards. Simultaneously, a novel reward-period response appeared during the new food-delivering phase (5.0–7.0s). We found no cue3 activity in all periods. This neuron was thus expected to code for the actual reward in the reward period. The actual reward model gave rise to the smallest AIC (inlet table), thus confirming the status of type-1 neuron. In contrast, the tonic cue/peck period activity remained in the omission (delay) block, thus supporting the idea that the neuron also coded the reward prediction prior to the food delivery. However, the lack of a delay-period response conflicts with the simulated target signal $r_{t}+\gamma \hat{V}\left(S_{t}\right)$ (Figure 2).

Figure S8A shows the averaged firing rates of 10 excitation subtype type-1 neurons. The data for neurons recorded in the omission and delay conditions were grouped together for the period before *t* = 5 s (left figure) and grouped separately afterwards (right figure). The rapid drop in reward-period activity was reproduced (Figure 7C). We thus expected these neurons to code the actual reward in the reward period, and to code the reward prediction in the cue/peck period. However, the lack of response in the delay period conflicts with the simulated $r_{t}+\gamma \hat{V}\left(S_{t}\right)$ signal (Figure 2).

#### Type-2 neurons

##### Omission condition

Figure 7D shows a representative example. In the control block, two tonic responses appeared in the cue/peck period and the reward period. In the omission block, the sign of the reward-period activity changed immediately from excitatory to inhibitory. Unlike our observations regarding the MSt type-2 neurons (Figure 4E), we observed little or no cue3 activity in the reward period. Thus, this neuron was expected to code the negative prediction error in the reward period. The prediction error model gave rise to the smallest AIC (inlet table), thus confirming the status of type-2 neuron. In contrast, in the omission block, the tonic cue/peck period activity remained while the delay period activity was inhibited. We did not observe any cue3 activity prior to reward delivery. With the exception of the excitatory reward-period response for cue1 in the control block, all features fit the simulated TD-error signalδ_*t*_ well (Figure 2).

##### Delay condition

Figure 7E shows a representative example. In the control block, a strong tonic response appeared in the cue/peck period, while a weak tonic response appeared in the reward period. In the omission (delay) block, the sign of the reward-period activity changed immediately from excitatory to inhibitory. Simultaneously, a novel but weak reward-period response appeared during the new food-delivering phase (5.0–7.0 s). We observed a weak transient cue response to cue3 but no response in the other periods. This neuron was thus expected to code the negative prediction error in the reward period. The prediction error model gave rise to the smallest AIC (inlet table), thus confirming the status of type-2 neuron. Conversely, the tonic cue/peck period activity remained in the omission (delay) block, although the amplitude declined slightly. These features fit the simulated TD-error signal δ_*t*_ well (Figure 2).

Figure S8B shows the averaged firing rate of 4 type-2 neurons. The rapid drop and inverted sign were reproduced (Figure 7F). The response patterns of these neurons are in concert with the simulated δ_*t*_ signal (Figure 2).

#### Type-3 neurons

##### Omission condition

Figure 8A shows the only neuron observed in this category. In the control block, an inhibitory tonic response appeared from cue1 onset and continued until the end of the reward period. In the omission block, the reward-period response remained and gradually disappeared. In the reacquisition block, the attenuated response quickly recovered when food delivery was reinstated. We did not observe any cue3 activity in the reward period. This neuron was thus expected to code the reward prediction in the reward period. The reward prediction model gave rise to the smallest AIC (inlet table), thus confirming the status of type-3 neuron. Conversely, the cue/peck/delay period activity remained in the omission block, with nearly the same amplitude. We also found a response to cue3 in the cue/peck/delay periods, but the amplitude was weaker. These features support the idea that the neuron coded the reward prediction prior to the food delivery. With the exception of the clear cue-period response, this neuron fits the simulated prediction signal $-\hat{V}\left(S_{t-1}\right)$ well (Figure 2).

##### Delay condition

Figure S9 shows a representative example and Figure S10 shows the averaged firing rate from two neurons. In the control block, these neurons showed responses in the cue period and reward period. In the omission (delay) block, the reward-period response remained and gradually disappeared. These responses were rather noisy, so we have chosen not to discuss the details of the firing patterns. Given the lack of a delay period response, we argue that the activity of these neurons conflicts with that of the simulated $\hat{V}\left(S_{t-1}\right)$ signal (Figure 2).

### Linear summation model of the TD error signal

As an additional post-categorization analysis, two-way ANOVA was applied to examine the differences among the three types of neurons in their normalized firing rate in the cue1 trials of omission block (Figure S12). These types generally showed significantly different firing rates, except that the difference between type-1 and type-3 MSt neurons (excitation subtype) were not statistically different. It is thus suggested the recorded set of neurons formed a continuum spectrum, rather than distinct groups separated by gaps, similarly to those shown in recent comparable study in mice (Tian et al., 2016).

As detailed above, type-1 neurons in the MSt (excitation subtype; Figures 4A,B) appear to code the target signal $r_{t}+\gamma \hat{V}\left(S_{t}\right)$. In contrast, type-3 neurons in the MSt (inhibition subtype; Figures 5C,D) may code the prediction signal $-\hat{V}\left(S_{t-1}\right)$. We examined whether these two populations of neurons could sufficiently account for the TD-error signal δ_*t*_ of type-2 neurons in the tegmentum (Figures 7D,E; Figure S8B). To this end, we constructed a simple model of linear summation. The averaged z-score of excitatory type-1 neurons in the MSt was assigned as *AR*_*str*_ (actual reward in the striatum, target signal). Similarly, the averaged z-score of inhibitory type-3 neurons in the MSt was termed *RP*_*str*_ (reward prediction in the striatum, prediction signal). We expected the weighed sum of these two values to yield the z-score of type-2 neurons in the tegmentum (*PE*_*teg*_, TD-error) as expressed by:

(4) $$\begin{array}{l} {PE}_{teg}=\;\beta_{1}\cdot {AR}_{str}+\;\beta_{2}\cdot {RP}_{str} \end{array}$$

**Figure 9:**
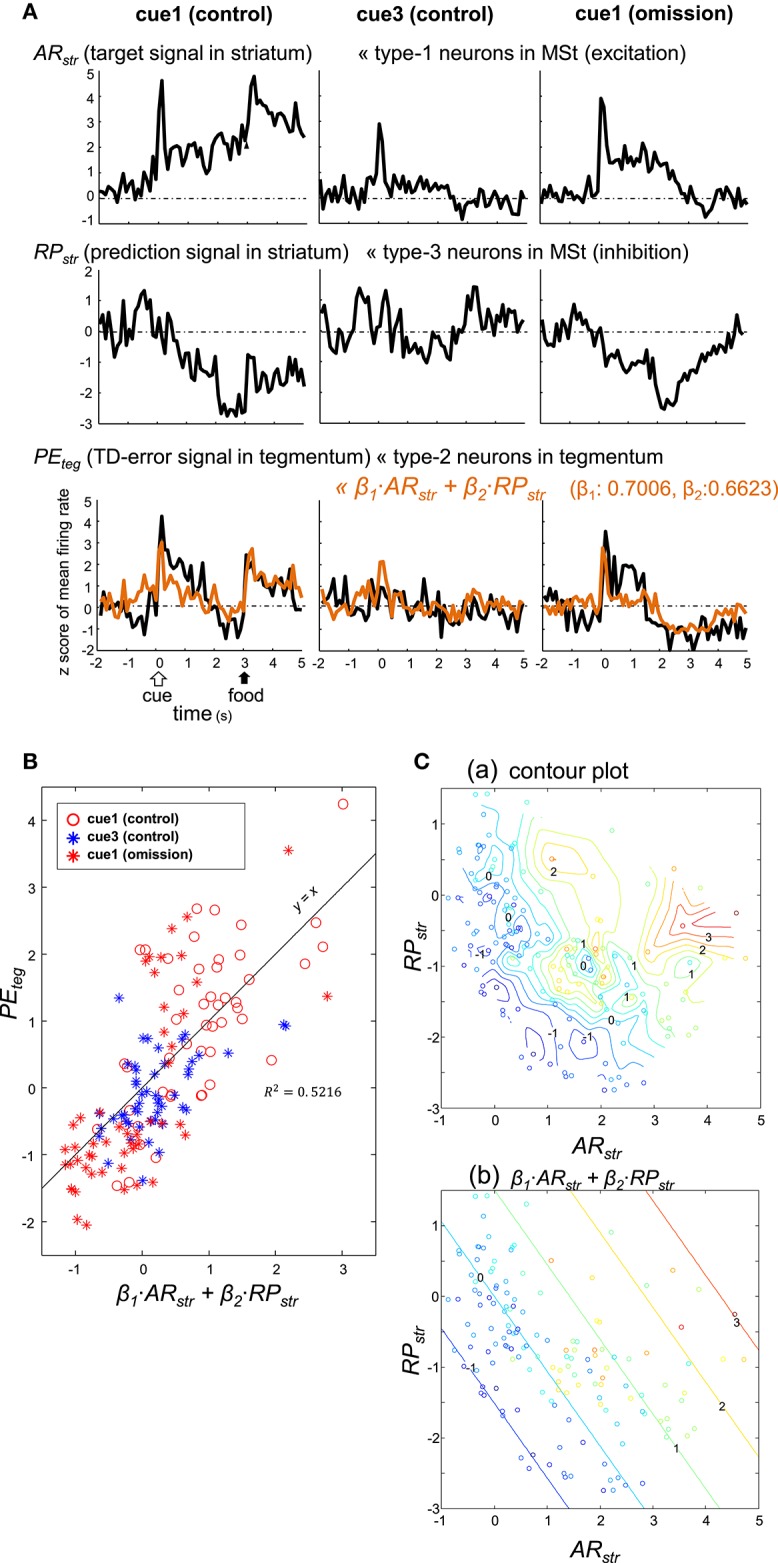
**Type-2 neuronal activity in the tegmentum (presumptive TD-error signal, PE_*teg*_) fitted as a linear sum of the activities observed in a subset of MSt neurons. (A)** Averaged activity of type-1 (AR_*str*_) and type-3 (RP_*str*_) MSt neurons, shown together with that of type-2 tegmentum neurons (PE_*teg*_) for 3 blocks of trials, cue1 (control), cue3 (control), and cue1 (omission). Superimposed orange lines on the PE_*teg*_ signal denote the linear sum of AR_*str*_ and RP_*str*_. **(B)** Scatter plot of PE_*teg*_ versus the linear sum. **(C)**. Pseudo 3-D plots of AR_*str*_ (x-axis), RP_*str*_ (y-axis), and PE_*teg*_ (color code) with the interpolated contour plot **(a)** and linear summation **(b)**.

Coefficients (β_1_ = 0.7006, β_2_ = 0.6623) were estimated using the least squares method without assuming a constant term. The linear sum (orange) was superimposed on the *PE*_*teg*_ (black) in the bottom traces of Figure 9A. Although the sum slightly underestimated the cue/peck period activities, it fit fairly well with the recorded *PE*_*teg*_ signal. Here, the trial period (from 0 to 5 s) was composed of 50 bins, as the bin width was 100 ms. For each of the trial types, the *PE*_*teg*_ value of these 50 bins (*y*-axis) were plotted against the corresponding sum (*x*-axis) in Figure 9B, with a considerable degree of correlation *R*^2^ = 0.5216. The paired *PE*_*teg*_ value was also color-plotted on a *AR*_*str*_ vs. *RP*_*str*_ plane in Figure 9C; contour plot in **C(a)**, and linear plot in **C(b)**. Discrepancies between these two plots indicate that the linear model was limited to a first-order approximation.

Alternatively, the *PE*_*teg*_ signal may be an appropriate fit for the sum of tegmental neurons, namely excitatory type-1 neurons (as *AR*_*teg*_) and the inhibitory type-3 neuron (as *RP*_*teg*_), as expressed by:

(5) $$\begin{array}{l} {PE}_{teg}=\;{\beta^{\prime }}_{1}\cdot {AR}_{teg}+\;{\beta^{\prime }}_{2}\cdot {RP}_{teg} \end{array}$$

**Figure 10:**
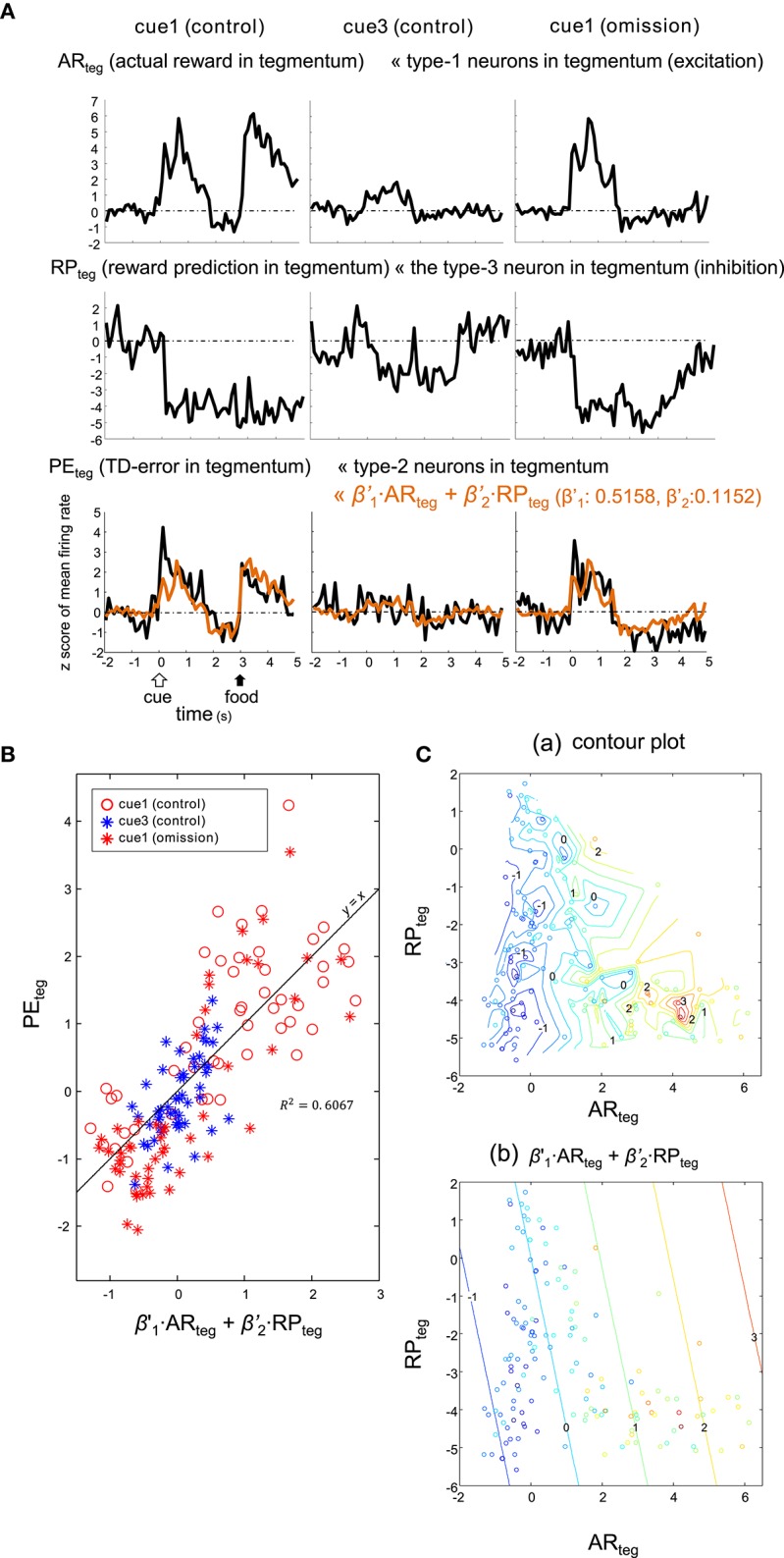
**Type-2 neuronal activity in tegmentum (presumptive TD-error signal, PE_*teg*_) fitted as a linear sum of the activities observed in a subset of the tegmentum neurons. (A)** Averaged activities of the type-1 (AR_*teg*_) and type-3 (RP_*teg*_) tegmentum neurons are shown together with those of the type-2 tegmentum neurons (PE_*teg*_) for 3 blocks of trials, cue1 (control), cue3 (control) and cue1 (omission). Superimposed orange lines on the PE_*teg*_ signal indicate the linear sum of AR_*teg*_ and RP_*teg*_. **(B)** Scatter plot of PE_*teg*_ versus the linear sum. **(C)**. Pseudo 3-D plots of AR_*teg*_ (x-axis), RP_*teg*_ (y-axis) and PE_*teg*_ (color code) with the interpolated contour plot **(a)** and linear summation **(b)**.

Here, the coefficients were estimated as: ${\beta^{\prime }}_{1}=0.5158$ and ${\beta^{\prime }}_{2}=0.1152$. This model (5) fit similarly to (4) (Figure 10), although the correlation for this model (*R*^2^ = 0.6067) was slightly higher. Thus, both striatal and tegmental representations of the reward and its prediction could be involved in the computation of TD error.

### Reciprocal connections between MSt and tegmental DA-ergic neurons

**Figure 11:**
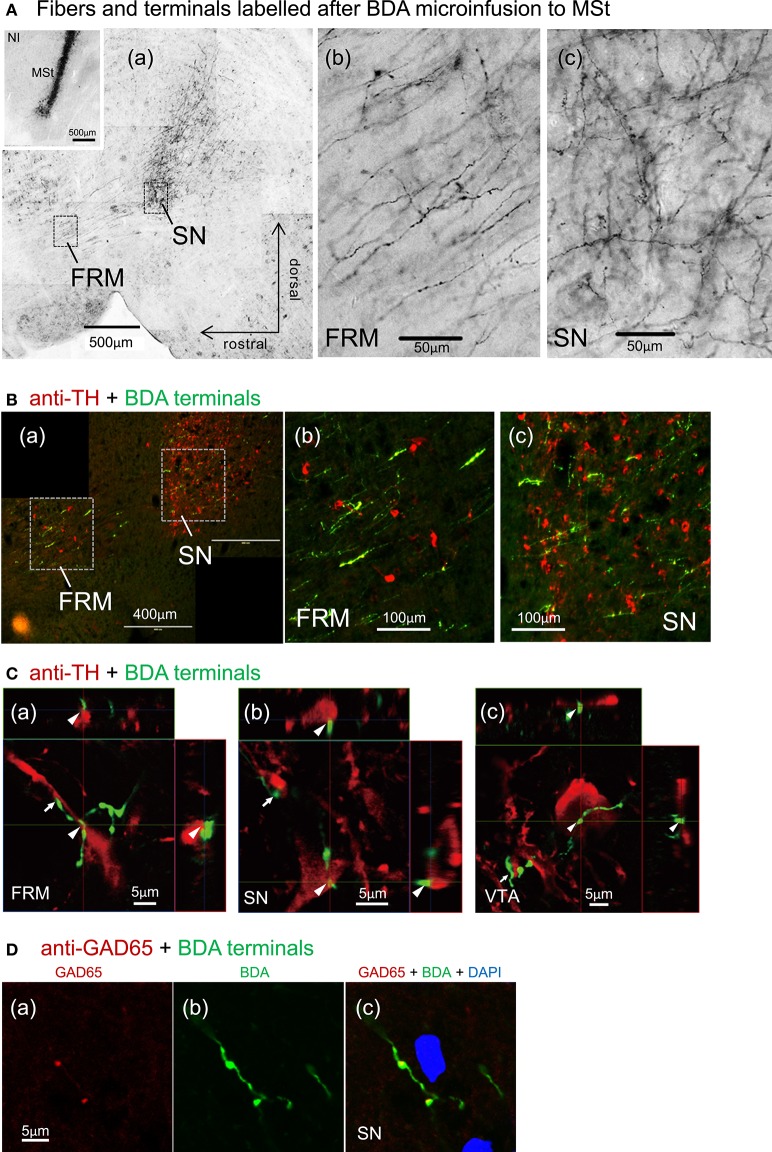
**Direct contacts of MSt terminals on DA-ergic neurons in the tegmentum. (A)** Dense arborizations of MSt efferent fibers were found in the FRM and SN (sagittal plane); low magnification **(a)**, high magnification in the FRM **(b)**, and the SN **(c)**. The inlet figure shows the injection site in the MSt. **(B)** BDA/TH double labeling in the tegmentum; BDA, green; TH, red. **(C)** Confocal images of direct contacts between BDA-positive terminal boutons and TH-positive dendrites and soma. Reconstructed on 3 orthographic planes. Arrowheads and arrows indicate the close appositions. **(D)** BDA/GAD65 double labeling in the SN, indicating co-localization on the terminal boutons. Sagittal sections with laterality: L1.4 in **(Aa–c)**; L0.9 in the **(A)** inlet; L1.4 in **B(a–c)**; L1.5, 1.1, and 1.0 in **C(a–c)**; L1.3 in **D(a–c)**.

After micro-infusion of BDA to the MSt, we found dense anterogradely labeled fibers in the FRM and SN of the ipsilateral tegmentum (Figure 11A), and less dense fibers in the VTA (not shown). Branching fibers and varicosities (**A(b, c)**) indicate the presence of MSt neuron synaptic terminals in the FRM and SN. BDA and anti-TH double labeling indicated a high degree of overlap between MSt terminals and DA-ergic neurons in the tegmentum (Figure 11B). High magnification observation using confocal microscopy revealed close apposition (arrowheads and arrows) between the MSt terminals (green) and the TH-positive neurons and proximal dendrites (red) in the FRM, SN, and VTA (Figure 11C). Some cases of varicosity in BDA positive terminal boutons were co-localized with instances of anti-GAD65 labeling, indicating that some MSt terminals are GABA-ergic (Figure 11D).

**Figure 12:**
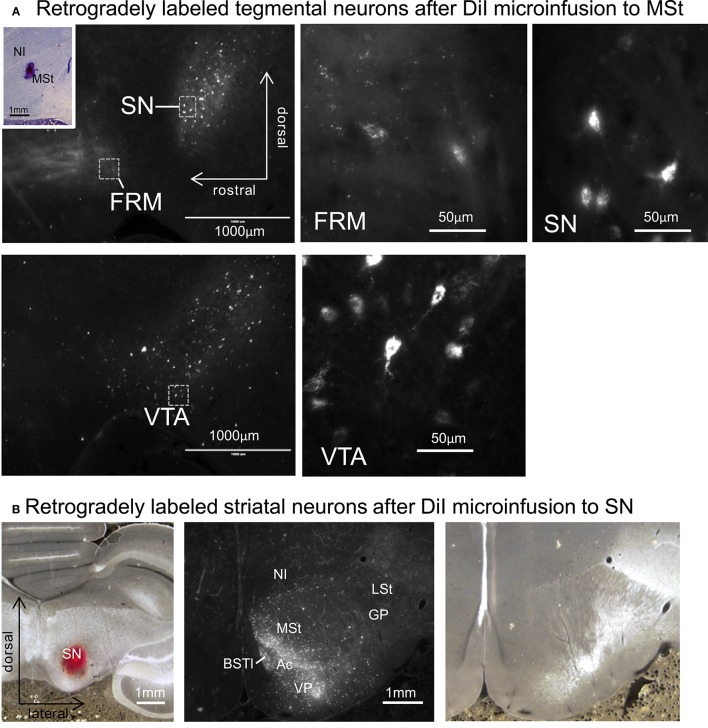
**Reciprocal projections between the MSt and tegmentum. (A)** Retrogradely labeled neurons in the FRM, SN, and VTA after micro-infusion of DiI into the MSt. The inlet indicates the injection site. **(B)** Retrogradely labeled neurons in the striatum after DiI infusion into the SN (left). DiI image (middle) and corresponding bright-field photo (right). See Appendix Supplementary Material for abbreviations. Sagittal planes with laterality: L1.2 in the injection site, L1.4 in the FRM and SN, and L0.8 in the VTA. Frontal planes with A-P level: A3.4 in the injection site, A9.4 in the striatum.

After micro-infusion of DiI to the MSt, we found retrogradely labeled cell bodies in the FRM, SN, and VTA (Figure 12A). The projection neurons were dense in the SN and VTA, while we only found a few neurons in the FRM. When DiI was injected into the SN, we found retrogradely labeled neurons in several areas in the ipsilateral striatum, such as the MSt, Ac, BSTl, and VP (Figure 12B). The medial part of the MSt contained more labeled neurons than the lateral MSt, suggesting a functional separation between these two sub-regions.

## Discussion

### Striatal representations of the target signal and the prediction signal

The neuronal mechanisms involved in the computation of TD error have been intensively studied. The mechanisms for one TD method, termed the actor-critic method, have been localized in the basal ganglia (Barto, 1995; Houk et al., 1995). Specifically, DA-ergic neurons, together with striatal neurons, have been assumed to play a critical role as the “critic” in the computation of TD error (Houk et al., 1995; Joel et al., 2002; Doya, 2007). Several lines of supporting evidence have been developed in mammals and birds. First, the striatum provides one of the major projections descending to DA-ergic neurons in the tegmentum (Anderson et al., 1991; Mezey and Csillag, 2002; Watabe-Uchida et al., 2012). Second, localized lesion and pharmacological manipulation studies have reported critical involvement of the striatum in reinforcement learning (Annett et al., 1989; Izawa et al., 2001; Ichikawa et al., 2004; Clarke et al., 2008; Rueda-Orozco et al., 2008; Castañé et al., 2010; Ogura et al., 2015). Third, during reinforcement tasks, striatal neurons show reward-related activities both before and after mammals (Tremblay et al., 1998; Janak et al., 2004; Apicella et al., 2009; Kim et al., 2009) and birds (Yanagihara et al., 2001; Izawa et al., 2005; Amita and Matsushima, 2014) receive a reward.

**Figure 13:**
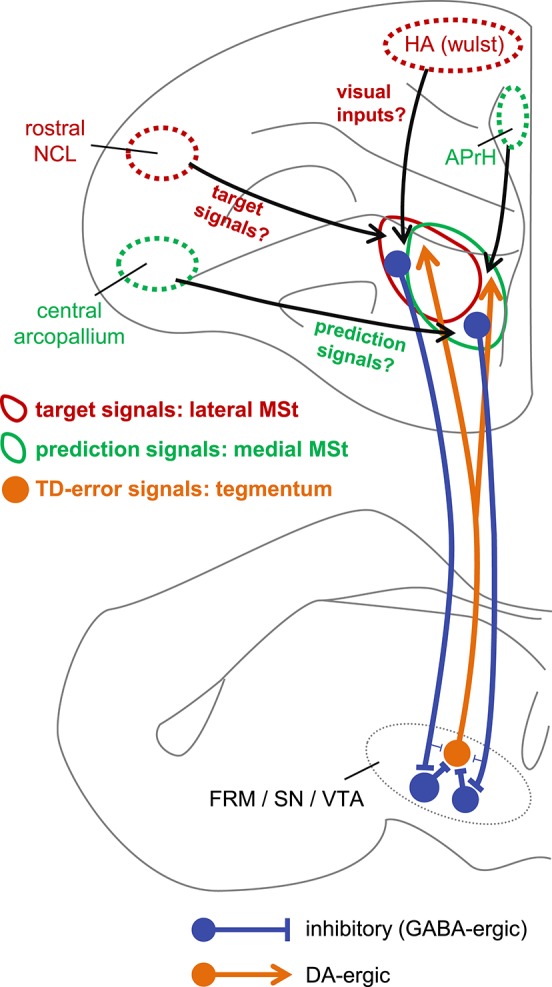
**Hypothetical neuronal mechanism for TD error computation**. The target signal and the prediction signal are coded by different but anatomically overlapping populations of neurons in the lateral MSt (solid red circle) and medial MSt (solid green circle), respectively. These two signals are sent to the tegmentum (FRM/SN/VTA) by GABA-ergic projection neurons (blue). In the tegmentum, these two signals converge via inhibitory local GABA-ergic inter-neurons. Through this convergent disinhibition, the TD error signal appears in DA-ergic neurons (orange filled circle). The DA-ergic neurons project back to a wide range of striatal areas, including the MSt (orange line arrow). In contrast, the lateral and medial MSt receive inputs from different pallial regions, such as the central arcopallium, rostral NCL, HA (Wulst), and APrH in the hippocampal complex. Red circles (HA and rostral NCL) indicate regions that project mainly to the lateral MSt, while green circles (central arcopallium and APrH) indicate regions that project mainly to the medial MSt.

Despite the above-mentioned efforts, the detailed mechanisms of TD error computation have not been fully elucidated at the neuronal level. To the best of our knowledge, the present results suggest, for the first time, that two critical signals of TD learning are represented by striatum neurons. Based on our present results, Figure 13 illustrates our proposed neuronal network underlying TD error computation. The sign-inverted signal of the predicted value of the state *S*_*t*−1_ represents the prediction $-\hat{V}\left(S_{t-1}\right)$. In other words, the striatum retains the reward prediction signal even after the food is delivered. The signal $r_{t}+\gamma \hat{V}\left(S_{t}\right)$ represents the target of $\hat{V}\left(S_{t-1}\right)$. Through the course of learning, the prediction signal $\hat{V}\left(S_{t-1}\right)$ approaches the target signal $r_{t}+\gamma \hat{V}\left(S_{t}\right)$ according to the difference between these two signals, i.e., the TD-error signal.

The prediction signal that we observed in chick striatal neurons (Figure 5D) is similar to those found in the GABA-ergic neurons of the mouse VTA (Cohen et al., 2012). Mouse GABA-ergic neurons were found to code prediction in the reward period. The firing gradually increased after the onset of a reward-predictive cue, and sustained even after the reward was received. The activity in the reward period remained unaltered even in omission trials. Similar neuronal signals have been reported in the striatum in mammals (Tremblay et al., 1998; Kim et al., 2009; Oyama et al., 2015). Note, however, that in the study by Kim et al. (2009), the researchers expected the neurons to code the action value rather than the state value. See below for discussions on the distinction between these two forms of value representation.

The target signal $r_{t}+\gamma \hat{V}\left(S_{t}\right)$ found in this study has two components, i.e., (1) the actual reward *r*_*t*_, and (2) the expected value of the current state $\gamma \hat{V}\left(S_{t}\right)$. Similar *prediction followed by reward* activity has been reported in monkeys (Tremblay et al., 1998), although this finding has not been associated with TD learning theory. Instead, we suggest that the same neuron may represent these two components as a critical signal in TD learning.

In this respect, it is worth noting that type-1 and type-3 neurons differed in terms of recording site (Figure 3A). The type-3 neurons (the prediction signal) were found in the medial part of the MSt and the Ac, whereas the type-1 neurons (the target signal) were located in the lateral part of the MSt. In a neuroanatomical study in pigeons, the medial part of the MSt was found to receive afferents from several pallial regions (Veenman et al., 1995). Of these, two regions are important in reinforcement learning, i.e., the central arcopallium and the prehippocampal area (APrH). Some neurons in the central arcopallium showed sustained responses during reward omission (Aoki et al., 2003), similar to the prediction signal found in the MSt. The APrH is thought to be analogous to the mammalian cingulate cortex (Veenman et al., 1995), which also codes actual reward, prediction, and prediction error in monkeys (Seo and Lee, 2007). Thus, these two regions may supply the prediction signal to the medial MSt.

In contrast, the lateral part of the MSt receives inputs from other pallial regions (Veenman et al., 1995; Kröner and Güntürkün, 1999). Of these, two regions may be critical. The first is the apical part of the hyperpallium (HA), which is part of the Wulst, one of the major visual centers (Ocklenburg and Güntürkün, 2012). The HA may supply the lateral MSt with the necessary information regarding color cues and food. The second critical region is the rostral part of the caudolateral nidopallium (rostral NCL). Neurons in the NCL show sustained responses to reward-predictive cues and responses to actual rewards (Diekamp et al., 2002), similar to the target signal found in this study in the MSt. These two regions may thus converge onto lateral MSt neurons, giving rise to the target signal.

### Striatal and tegmental representations of TD error

In the present study, we found that type-2 neurons in the MSt (Figures 4D–F) and tegmentum (Figures 7D–F) fit the model of prediction error better than the alternatives. In the omission block, both of these neurons showed (1) excitation in the cue/peck period, and (2) inhibition (lower firing below the baseline) in the following reward period. Both of these features match those assumed to be involved in the TD error signal. However, in the control block, these neurons showed excitation in the reward period. If chicks had been over-trained such that they accurately predicted the reward amount, such excitatory activity should not occur. Because of this conflict, we argue that the inhibition in the reward period found in the type-2 neurons may not represent the prediction error.

One possible explanation for the above finding is that the chicks were not fully trained to discriminate color cues. As shown in the behavioral data (Figure 1C), even after intensive training for 3 days or longer, the chicks still pecked cue3 (non-rewarding color cue) in 25–50% of the trials. Similarly, in the cue1 and cue2 trials, the chicks might not have predicted the food with 100% certainty. This may explain why the TD signal was positive in the reward period, in which food was only partially predicted. Indeed, DA-ergic neurons in monkeys showed a similar pattern of excitation in response to a predicted reward (Fiorillo et al., 2003; Morris et al., 2004).

An alternative explanation is that the type-2 neurons code the target signal $r_{t}+\gamma \hat{V}\left(S_{t}\right)$ rather than the TD error δ_*t*_, similar to the type-1 neurons. The inhibition observed during the reward period could be due to food omission, rather than the prediction error signal. This is particularly plausible in the striatal type-2 neurons (Figure 4E), in which similar inhibition occurred in the cases in which the omission of food was predicted (cue3: dashed blue line) and unpredicted (cue1: dashed red line).

However, it is not possible to explain the pattern of tegmental type-2 neuron activity in this manner, because distinct activities occurred in cue1 (omission/delay) and cue3 (control) trials (Figures 7D–E). Thus, this neuron type might fit the explanation that these code the TD-error. However, we had no evidence as to whether the tegmental type-2 neurons are DA-ergic neurons. In an electrophysiological study of zebra finches, DA-ergic neurons in the VTA and SNc (substantia nigra, pars compacta) exhibited wider spikes and a lower firing rate compared with non-DA-ergic neurons in the same regions (Gale and Perkel, 2006). In our present study, on the other hand, we found no significant differences in spike width and firing rate among the three neuron types (Figures 6B,C). Importantly, the prediction error signal in previous studies has also been found in non-DA-ergic neurons (Schultz, 2015), including those in the striatum in rats (Kim et al., 2009; Oyama et al., 2010) and monkeys (Apicella et al., 2009). The different firing patterns observed in the type-2 neurons in the MSt and tegmentum may imply that these regions have different functionality.

### Tegmental neurons may also contribute to TD-error computation

Summation of activities of the type-1 and type-3 neurons in tegmentum also yielded a fitting to the TD error signal (Figure 10). The correlation coefficient of the linear plot (Figure 10B) was comparable to that found for the striatal neurons (Figure 9B). The tegmental neurons could thus contribute, similarly to the local GABAergic neurons in the mouse VTA (Cohen et al., 2012; Eshel et al., 2015). However, we must notice that (1) the type-1 and type-3 neurons in the tegmentum did not fit well to the TD learning signals, and (2) the fitting was based only on one recorded type-3 neuron (Figure 8). Further surveys on the tegmental neurons are necessary.

In a very recent paper in mice Tian et al. (2016), that appeared after the submission of our present study), neuronal activities were recorded from neurons with confirmed monosynaptic connection to DA-ergic neurons. These input neurons were distributed widely in various brain regions including dorsal and ventral striatum, as well as lateral hypothalamus and tegmental nuclei. Interestingly, they found diverse sets of firing patterns in these regions, similar to those found in our present study (Figures 3–8). Tian et al. (2016) also reported those neurons that coded “pure reward,” “pure expectation,” or a mixture of both. In particular, a subset of striatal and tegmental neurons coded partial prediction error signal, similarly to our chick cases. Finally, a linear combination of inputs provided a good fitting of the reward prediction error signal represented by DA-ergic neurons, paralleling our linear summation model (Figures 9, 10). Despite the distinct evolutionary backgrounds between avian and mammalian brains, the mechanisms for TD error computation may be highly conserved.

### Neuroanatomical bases of TD error computation

#### Direct inhibitory pathway

Our tract-tracing experiments were consistent with previous reports regarding the connectivity between the MSt and the DA rich tegmentum nuclei in the avian brain. As previously reported in chicks (Székely et al., 1994), we confirmed that descending MSt neurons have direct synaptic contacts onto DA-ergic neurons in the FRM, SN, and VTA (Figure 11C). Our GABA immunostaining data (Figure 11D) also supported the previous finding that striatal projection neurons in pigeons are GABAergic (Reiner and Anderson, 1990). It is therefore reasonable to suggest that MSt projection neurons have an inhibitory effect on DA neurons. However, our hypothetical algorithm (Figure 2) and the linear summation model (Figure 9) assumes that excitatory type-1 and inhibitory type-3 MSt neurons have an excitatory effect on type-2 neurons in the tegmentum. Thus, how the descending inhibitory pathway mediates the summation of the two striatal signals in the tegmentum requires further explanation.

#### Indirect pathway for disinhibition

In addition to the direct inhibitory pathway, striatal neurons may indirectly affect DA-ergic neurons through local interneurons within the tegmental nuclei. A immuno-histochemical study in pigeons showed that DA-ergic neurons in the SN receive inputs from both SP-positive striatal neurons and SP-negative neurons, which may come from other regions (Anderson et al., 1991). The authors also reported that SP-positive striatal terminals contacted both DA-ergic and non DA-ergic neurons in the SN. A recent study in mice proposed the functional involvement of the indirect pathway, as nucleus accumbens neurons in the ventral striatum dis-inhibit DA-ergic neurons in the VTA by inhibiting GABA-ergic local inter-neurons (Bocklisch et al., 2013). Additionally, DA-ergic neuron activity in the VTA is suppressed by local GABA-ergic inter-neurons in mice (Eshel et al., 2015).

Similar disinhibitory action may occur in chicks. Our present tracing experiment is consistent with a previous study in chicks (Bálint et al., 2011), which reported that the above-mentioned DA-ergic tegmentum nuclei receive efferents from the MSt and Ac. However, it is important to know how and where the descending GABA-ergic inhibition is converted. As reported in mammals, candidates include the local GABA-ergic inter-neurons in the VTA, FRM and SN pars reticulate in the avian brain (Veenman and Reiner, 1994) (Figure 13). In future research, it will be critically important to determine whether the descending GABA-ergic MSt efferents have synaptic contacts with the presumed GABA-ergic local interneurons in the tegmental nuclei. Also, this disinhibition effect should be examined using electrophysiology.

### TD learning for updating state value and behavioral execution

#### Two types of TD errors for state value and action value

Generally, two different types of TD error signals have been studied using theoretical approaches (Sutton and Barto, 1998). The first type focuses on the TD of the state value. The classical actor-critic method adopts this type, which was assumed in the early studies of DA-ergic neurons (Montague et al., 1996; Schultz et al., 1997). Actually, neuronal activities in the ventral striatum and anterior cingulate of monkeys coded the progress of a task comprising a series of trials prior to a reward (Shidara et al., 1998; Shidara and Richmond, 2002). Thus, aspects of state may be coded in these regions. On the other hand, the second type focuses on the TD of the action value. Methods such as Q-learning and SARSA adopt this type of TD error. In recent studies, the second type also proved to be plausible, as DA-ergic neuron activity in a decision making task was accounted for by TD error via the SARSA (Morris et al., 2006) and Q-learning methods (Roesch et al., 2007). In the present study, we assumed the first type of TD error signals, and found neuronal activities that matched the simulated signals. Whether the second type is also implemented by striatal/tegmental neurons in decision making tasks is still unknown.

#### Representation of the action value in the striatum

In addition to reinforcement learning, the striatum is involved in the modulation of locomotor movements (Grillner et al., 2005). It is thus important to determine whether other striatal neurons code the action value, or the quality of several different actions (Sutton and Barto, 1998). As mentioned above, striatal networks may be critical for computing the second type of TD errors, and thus may code action value. Furthermore, the action value can guide the action selection in the actor-critic method (Barto, 1995), in which the action with a larger action value tends to be chosen more frequently. Action value signals have been found in the striatum in monkeys (Kawagoe et al., 1998; Samejima et al., 2005) and rats (Kim et al., 2009), and these may be modified by TD error signals issued by DA-ergic neurons (Doya, 2007). In the present study, chicks did not choose from multiple options, so we did not focus on the action value. As a future project, it will be important to determine whether striatal/tegmental neurons also code the action values for tasks in which subjects must choose from multiple targets or actions.

## Author contributions

CW and TM: Designed the experiment, interpreted the results and wrote the manuscript; CW: Performed the electrophysiological and tract-tracing experiments, developed the simulation and analyzed the data; YO: Performed the anti-TH staining, assisted the neuroanatomical experiments, and provided critical comments and revisions on the manuscript. All authors approved the final version of this manuscript.

## Funding

This study was supported by a grant funded to TM by the Ministry of Education, Science and Technology, and the Japan Society for the Promotion of Science (MEXT-JSPS Kakenhi) (Grant-in-Aid for Scientific Research #25291071 and Grant-in-Aid for Challenging Exploratory Research #26650114). YO was also funded by JSPS (Grant-in-Aid for JSPS Fellows, #26·8054).

### Conflict of interest statement

The authors declare that the research was conducted in the absence of any commercial or financial relationships that could be construed as a potential conflict of interest.

## Abbreviations of neural structures

Ac: nucleus accumbens
APrH: prehippocampal area
BCS: brachium colliculi superioris
BSTl: lateral part of the bed nucleus of the stria terminalis
EW: nucleus of Edinger-Westphal
nucleus nervi oculomotorii: pars accessoria (ICAAN)
FRM: formatio reticularis medialis mesencephalic
GP: globus pallidus
HA: apical part of the hyperpallium
Imc, nucleus isthmi: pars magnocellularis
LSt: lateral striatum
M: mesopallium
MSt: medial striatum
NI: nidopallium intermediate
NIII: nervus oculomotorius
NCL: caudolateral nidopallium
OMd, nucleus nervi oculomotorii: pars dorsalis
Omv, nucleus nervi oculomotorii: pars ventralis
OV: nucleus ovoidalis
PTM: nucleus pretectalis medialis
Ru: nucleus ruber
SN: substantia nigra
SP: nucleus subpretectalis
SpL: nucleus spiriformis lateralis
VP: ventral pallidum
VTA: ventral tegmental area.
