# Supplementary material for: Striatal and Tegmental Neurons Code Critical Signals for Temporal-Difference Learning of State Value in Domestic Chicks
Source: Front Neurosci. 2016 Nov 8;10:476. doi: 10.3389/fnins.2016.00476 (PMC5099172; doi:10.3389/fnins.2016.00476)
Supplement: Supplementary file 1 [file DataSheet1.pdf]

Supplementary material (contents)

Striatal and Tegmental Neurons Code Critical Signals for Temporal-Difference Learning of State Value in Domestic Chicks

Chentao Wen, Yukiko Ogura and Toshiya Matsushima

Text S1	Derivation of PE model	p.2
Table S1	Coordinates of the target regions	p.3
Figure S1	Waiting time during the early phase of the omission block	p.4-5
Figure S2	Pecking response during a long-term omission recording	p.6
Figure S3	TD learning simulated according to the task	p.7
Figure S4-1, 4-2, 4-3	Technical notes on single unit recording	p.8-10
Figure S5	Delay-period activity of a type-1 MSt neuron	p.11
Figure S6	Inhibitory type-1 neurons in MSt.	p.12
Figure S7	Inhibitory type-3 neuron in MSt	p.13
Figure S8	Population average of type-1 and type-2 tegmental neurons	p.13
Figure S9	Excitatory type-3 neuron in tegmentum	p.15
Figure S10	Average of type-3 neurons in tegmentum	p.16
Figure S11	Estimated learning rate (α) of individual neurons	p.17
Figure S12	Omission block activities compared among categorized groups of neurons	p.18-19

Text S1 Derivation of PE model

By definition:

$$pe_T = R_T - p_T \quad (1)$$

This is identical to:

$$p_T = R_T - pe_T \quad (2)$$

According to RP model (Appendix 4-2):

$$p_T = p_{T-1} + \alpha \cdot pe_{T-1} \quad (3)$$

By substituting p_T and p_{T-1} in (3) with (2), we get:

$$R_T - pe_T = R_{T-1} - pe_{T-1} + \alpha \cdot pe_{T-1} \quad (4)$$

To solve for pe_T, we get:

$$pe_T = R_T - R_{T-1} + (1 - \alpha) \cdot pe_{T-1}$$
$$= \Delta R + (1 - \alpha) \cdot pe_{T-1} \quad (5)$$

Target region	Distance (mm)			Angle between insertion and vertical line
	Anterior from bregma	Lateral from midline	Depth from brain surface	
MSt	1.5~2.0	1.25~1.5	5.0~5.5	leant 25° caudally
Anterior tegmentum	-0.5~1.0	0.9~2.75	6.8~9.0	14° rostrally
Posterior tegmentum	-3.3~-2.5	1.5	8.5~9.0	vertically

Table S1. The coordinates of the target regions for tetrodes implantations and tracer injection.

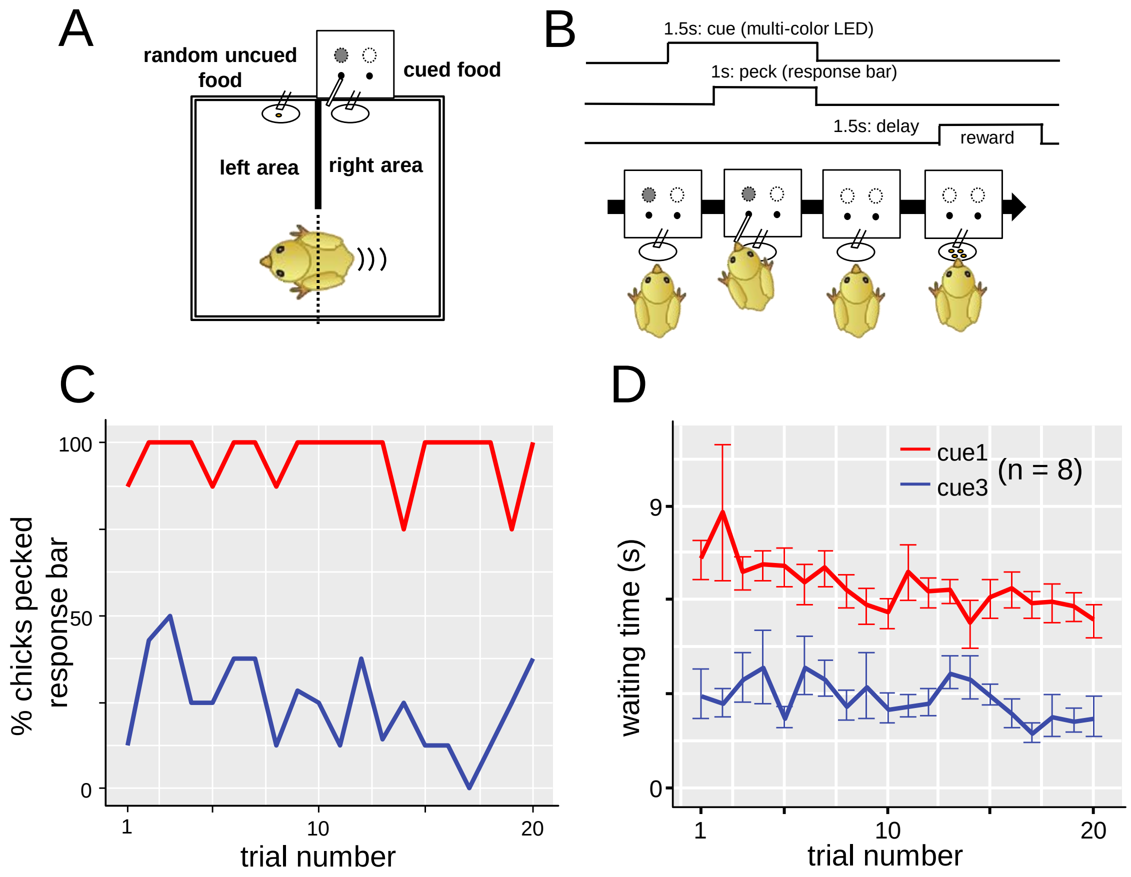

Figure S1. Decrease in the waiting time during the early phase of the omission block

Eight chicks were trained to associate color cues with reward exactly in the same manner as we have applied for electrophysiological experiments. (A, B) By an opaque wall, the chamber was partly separated into two areas (right and left), and the chick was allowed to freely walk between the two. Chicks were trained in the right area for the color-food associations, while a feeder at the left area randomly delivered food at a low frequency (1 grain of millet every 3 trials) without any sensory cues. The waiting time was determined as the period of time from the cue onset until the chick crossed the midline (as indicated by a dashed line) to the left area. Note that the food reward was delivered at 3.0 s after the cue onset. (C) The % chicks that pecked the response bar was plotted against the trial number (1 to 20 of omission block) for cue1 (S+, omission; red) and cue3 (S-; blue). (D) The waiting time in cue1 and cue3 trials plotted against the trial number. Mean ± SEM (n=8 chicks) are shown.

We fitted a linear mixed-effects model in which the waiting time was given by a linear summation of: *cue_type* (cue 1 or 3), *trial_number* (1 through 20), their *interaction* and

random effects for individuals. The following packages in R were used, "lme4" (Bates et al. 2015) and "lmerTest" (Kuznetsova et al. 2016). The results revealed significant effects of *cue_type* (t=6.817, df = 13.96, p < 0.001) and trial number (t=-2.401, df = 296.48, p < 0.02), but the interaction was not significant (t=-1.643, df = 149.95, p = 0.103 > 0.05).

Data shown in (C) reproduced the results shown in Fig. 1C. From data in (D), we conclude that (1) the waiting time was significantly longer in cue1 trials than cue3, (2) the waiting time decreased in both cue1 and cue3 trials, though slightly more in the cue1 trials. It is therefore appropriate to assume that chicks update the reward expectations during the reward period from the early phase of the omission block.

References:

Bates, D., Mächler, M., Bolker, B., and Walker, S. (2015). Fitting Linear Mixed-Effects Models Using lme4. *J. Stat. Softw.* 67, 1–48. doi:10.18637/jss.v067.i01.

Kuznetsova, A., Brockhoff, P. B., and Christensen, R. H. B. (2016). *lmerTest: Tests in Linear Mixed Effects Models*. Available at: https://CRAN.R-project.org/package=lmerTest.

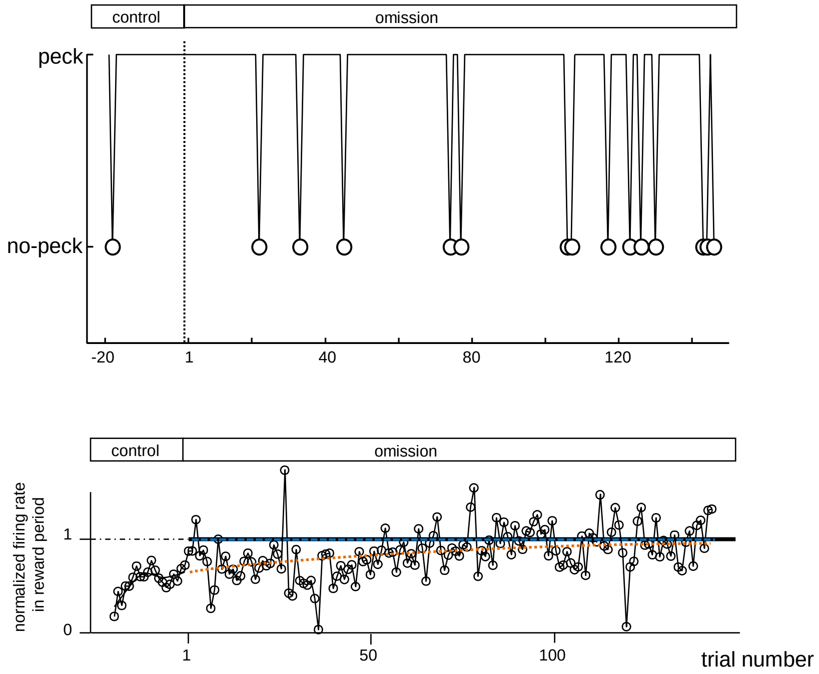

Figure S2. Example of a chick's pecking response in peck-period during a long term recording of omission block (same as Fig. 5C).

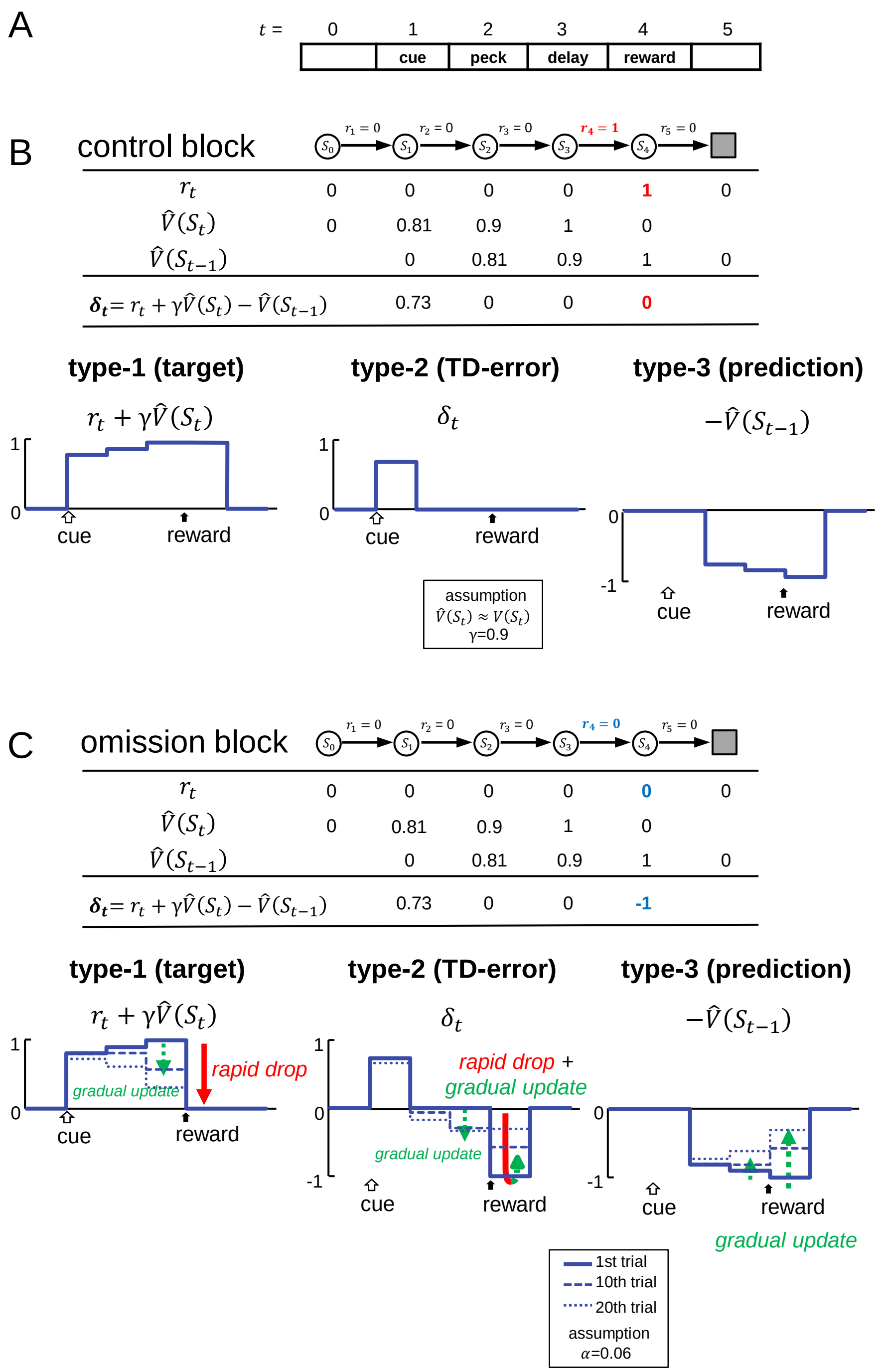

Figure S3. TD learning simulated according to the task ($\gamma = 0.9$ and $\alpha = 0.06$)

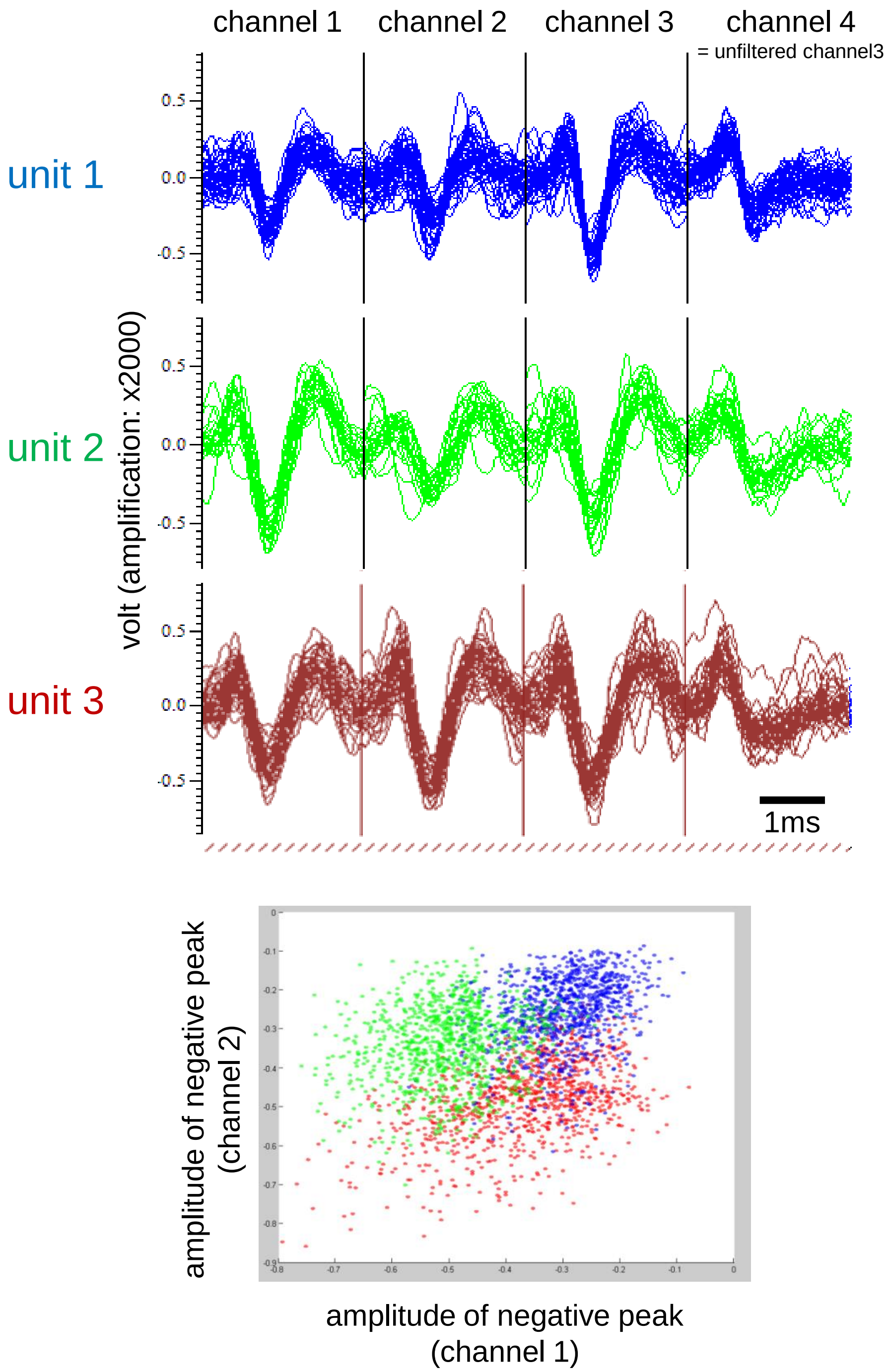

Figure S4-1. Example recording in which 3 separate neurons were encountered. Each unit was identified as the composite of four waves, and separated by waveform template matching. Simple amplitude plot of channel 1 and 2 data reveals a clear separation among the three simultaneously recorded neurons.

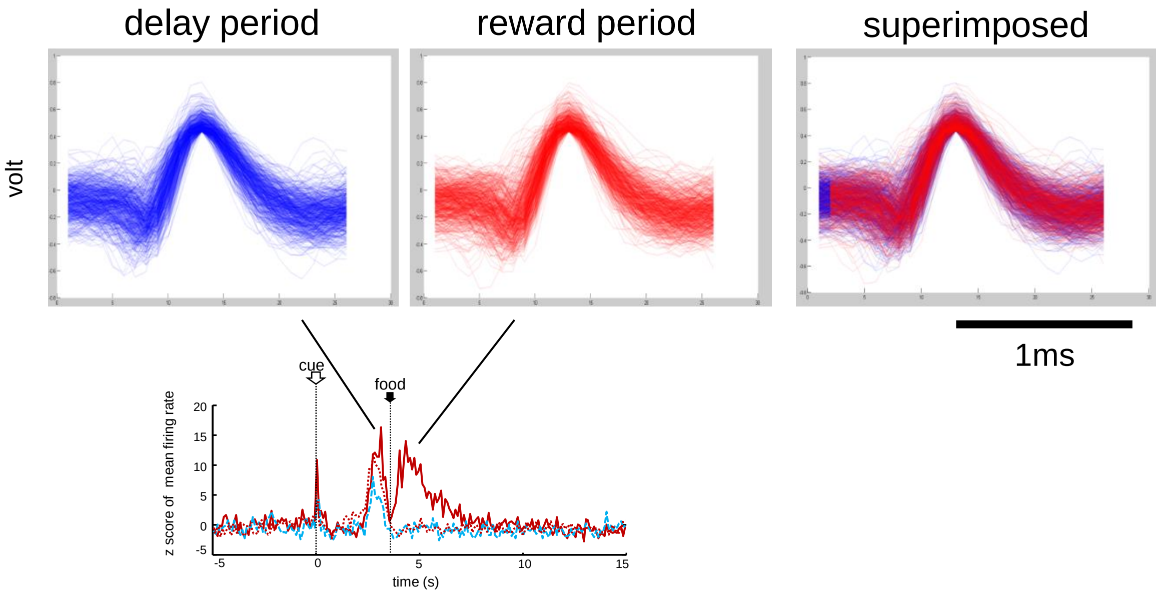

Figure S4-2. Action potentials recorded during the delay- and reward-periods perfectly matched, suggesting that these were issued from the same neuron.

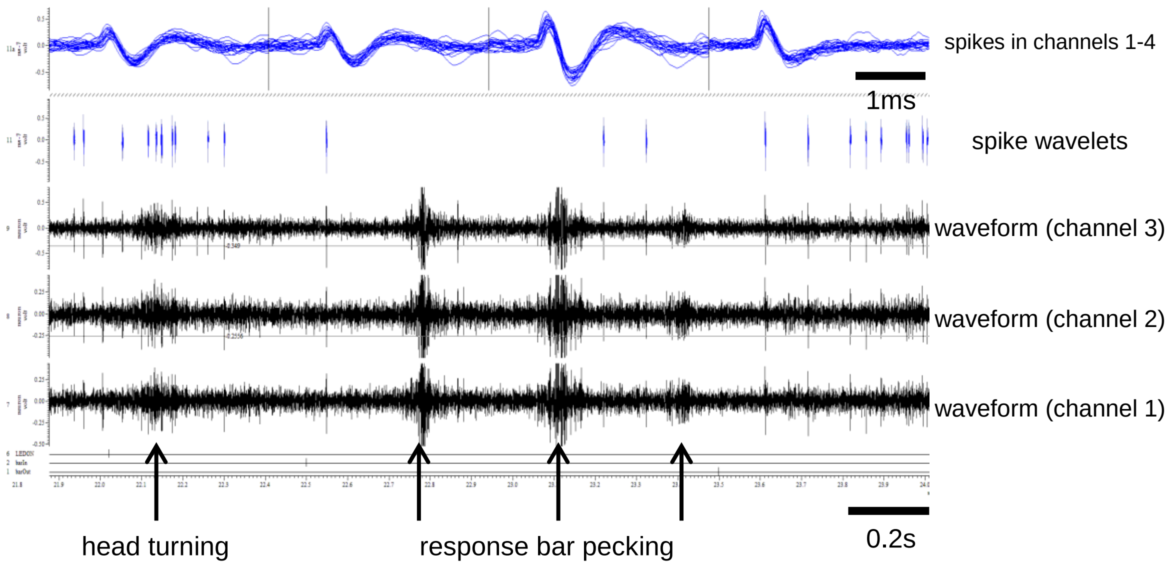

Figure S4-3. Examples of spike template recording and movement artifacts. Superimposed spikes (above) and chart recording (below). Spikes were separated from the movement artifact signals caused by head turning or response bar pecking. Burst of spikes coincided with the head turning artifact, but not the three bouts of artifacts at bar pecking.

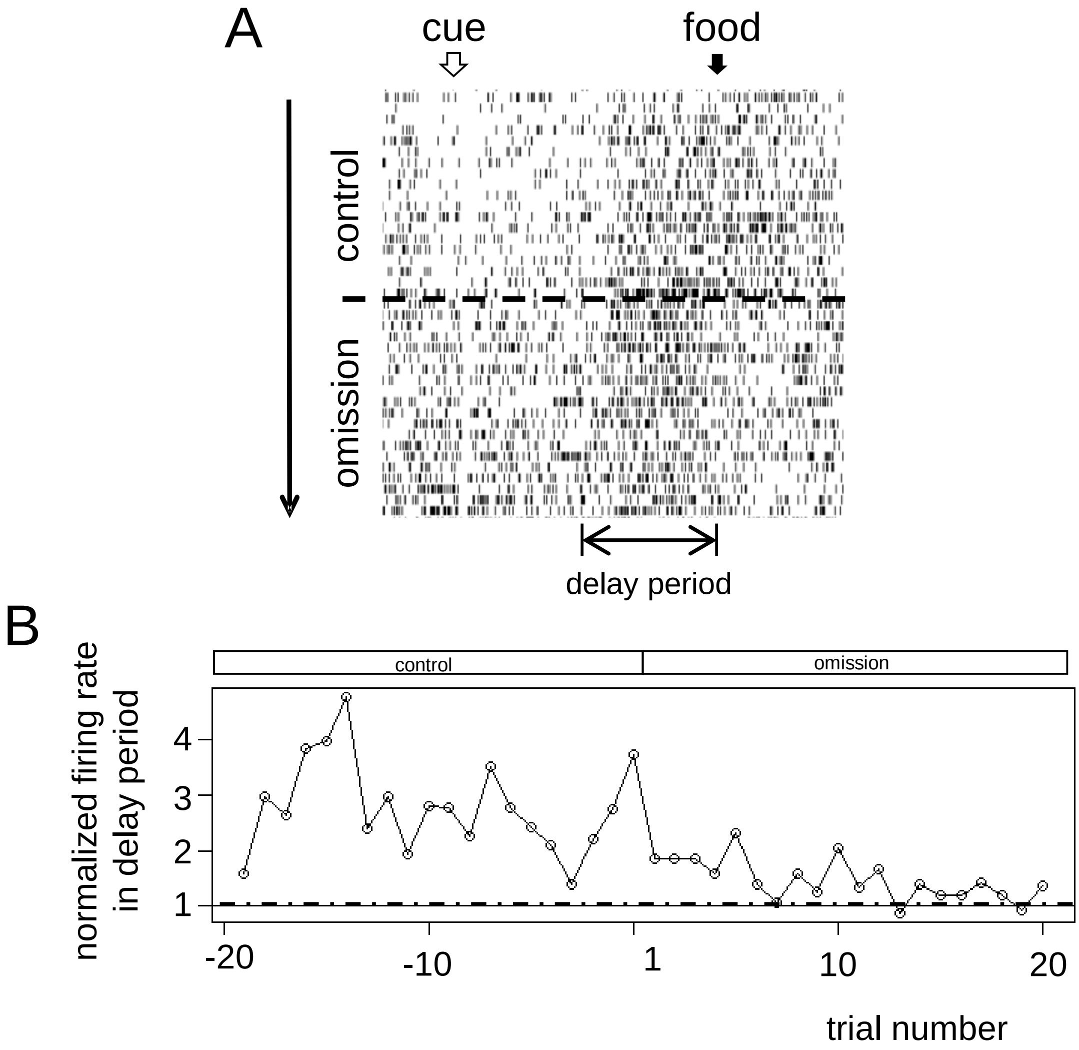

Figure S5. Delay-period activity of a type-1 MSt neuron. (A) Rastergram.(B) Normalized firing rate of the delay-period activities (1.5s~3.0s) is plotted against the trial number.

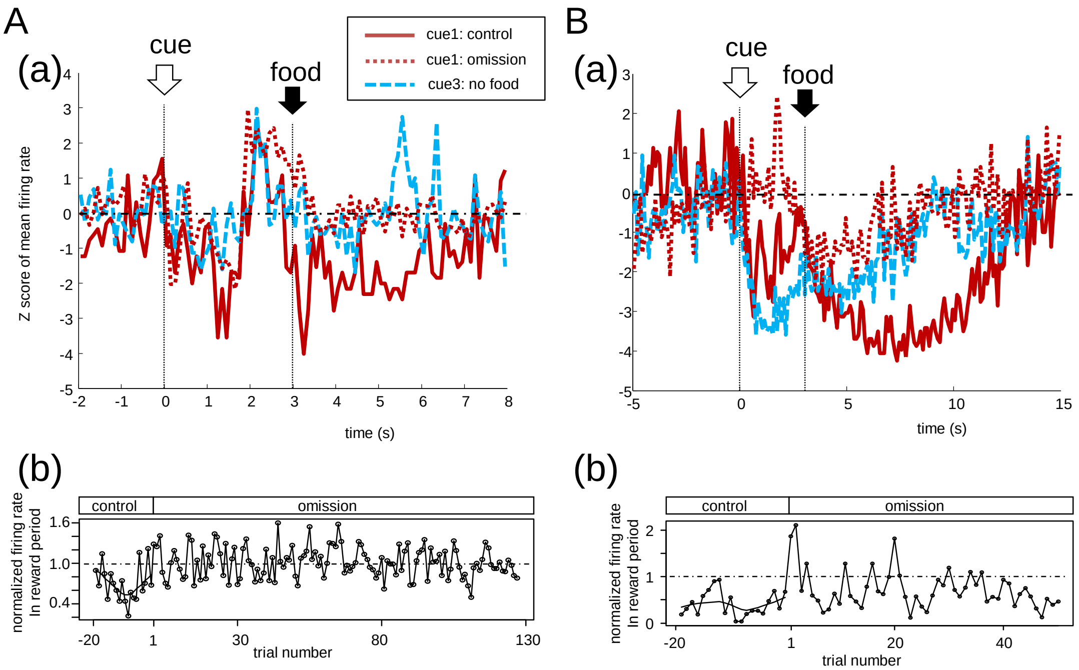

Figure S6. Two representative examples of the inhibitory type-1 neurons in MSt. (A) Neuron#78. (a) Averaged firing rate (z-score) in the cue1 trials is compared between the control block (red line) and the omission block (red dashed line).Data obtained in cue3 trials (blue dashed line) is superimposed. (b) Normalized firing rate in the reward period is plotted against the trial number. (B) Neuron #5. Conventions are as in (A). (a) Cue-/peck-/delay-period inhibition was followed by a further imbibition in the reward-period.

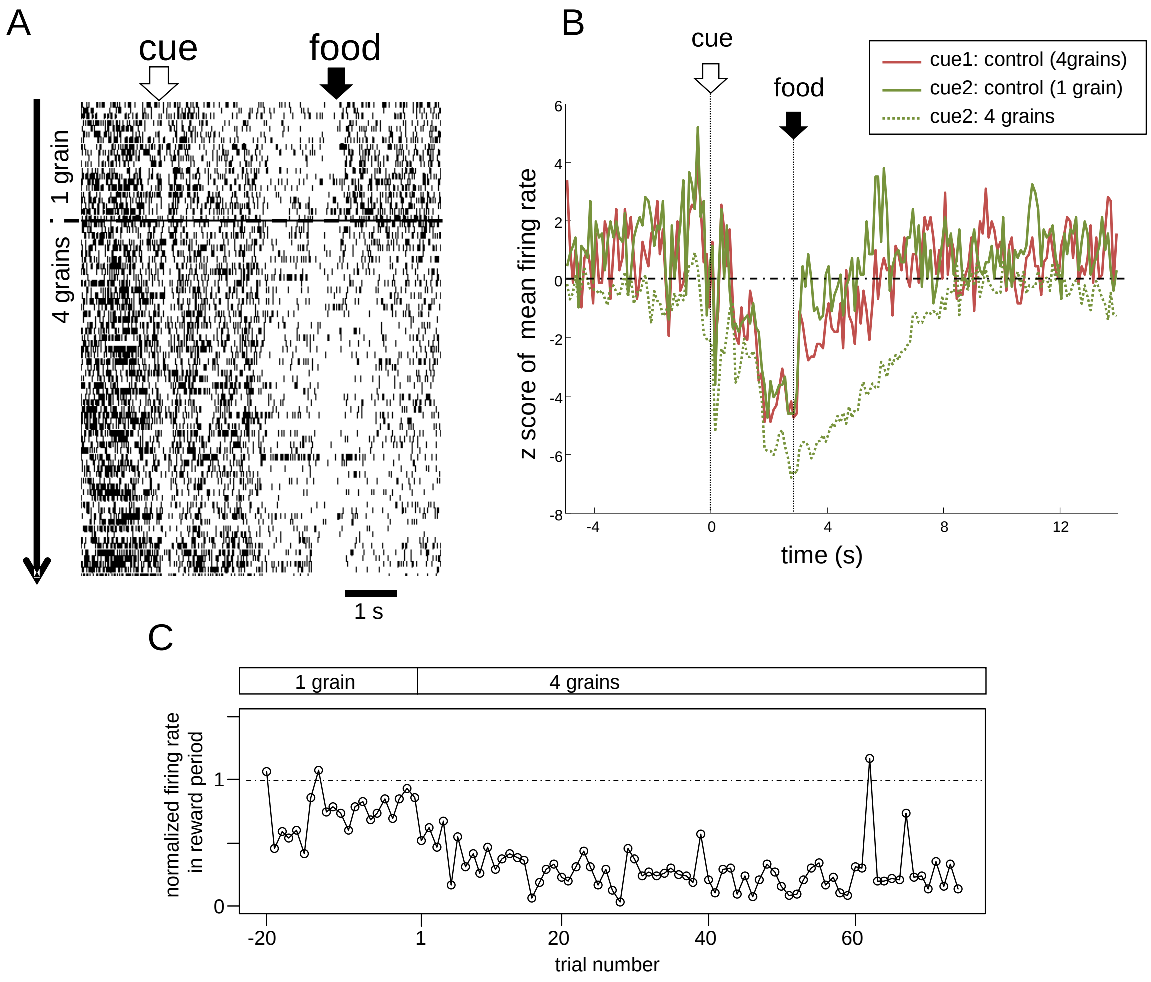

Figure S7. An example of inhibitory subtype of the type-3 neuron in MSt. In cue2 trials, reward increased from 1 grain to 4 grains. The same neuron as shown in Fig. 5C. After increase in the food reward, the amplitude of the reward-period activity gradually increased. (A) Rastergram of the cue2 trials. (B) Averaged firing rate (z-score). (C) Normalized firing rate in reward period of cue2 trials is plotted against the trial number.

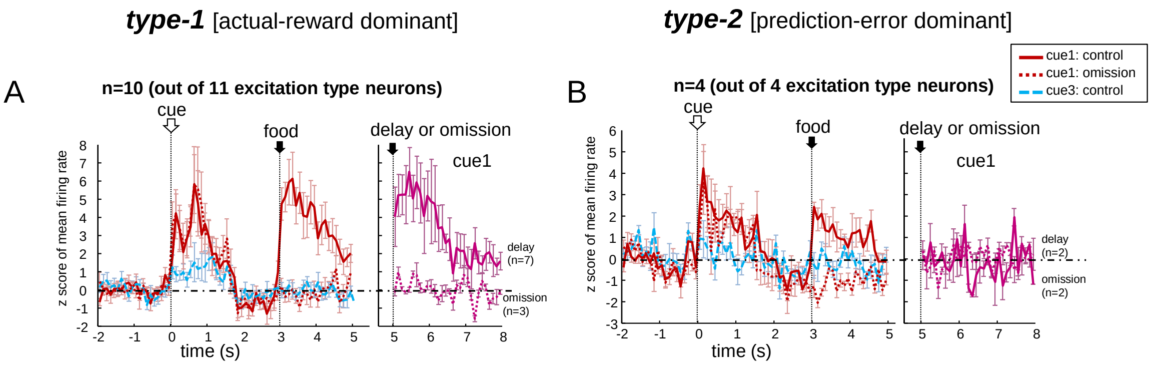

Figure S8. Population average of the type-1 (A) and type-2 (B) neuron activities in tegmentum.

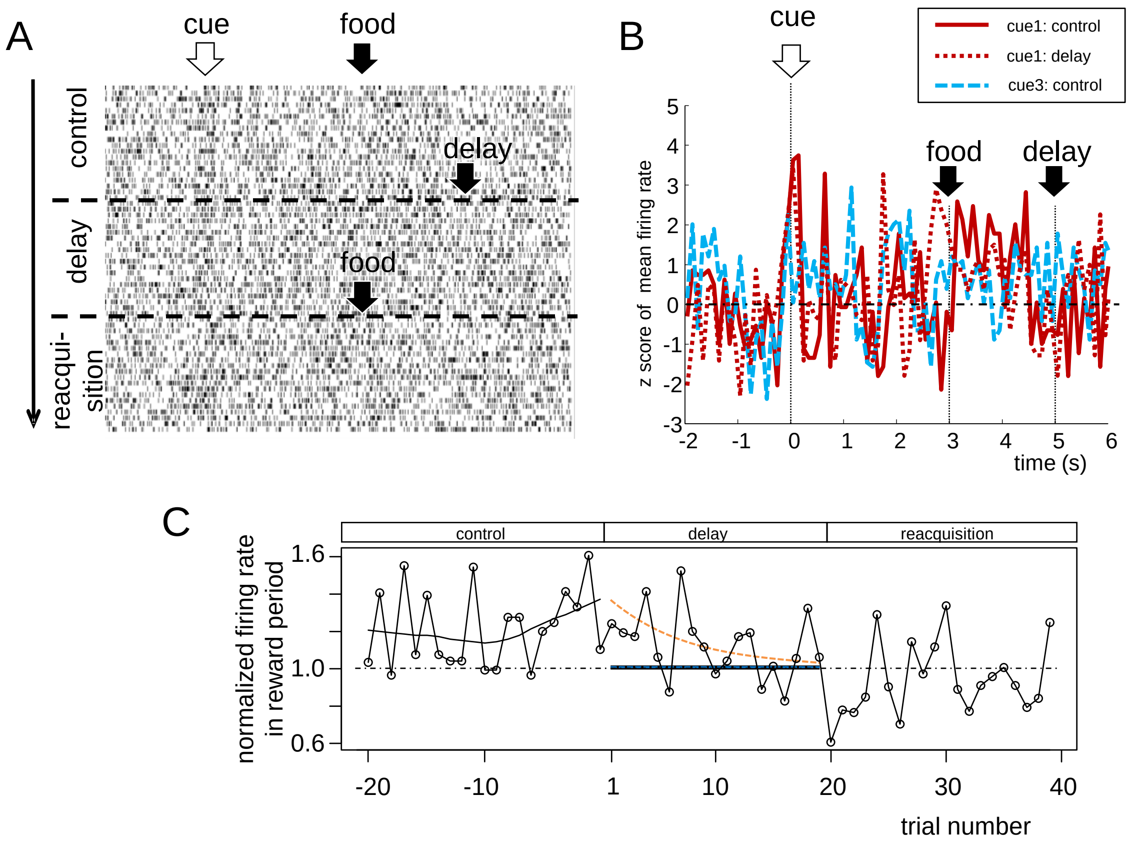

Figure S9. A representative example of excitatory subtype of the type-3 neuron in tegmentum. (A) Rastergram. (B) Averaged firing rate. (C) The reward-period excitation plotted against the trial number.

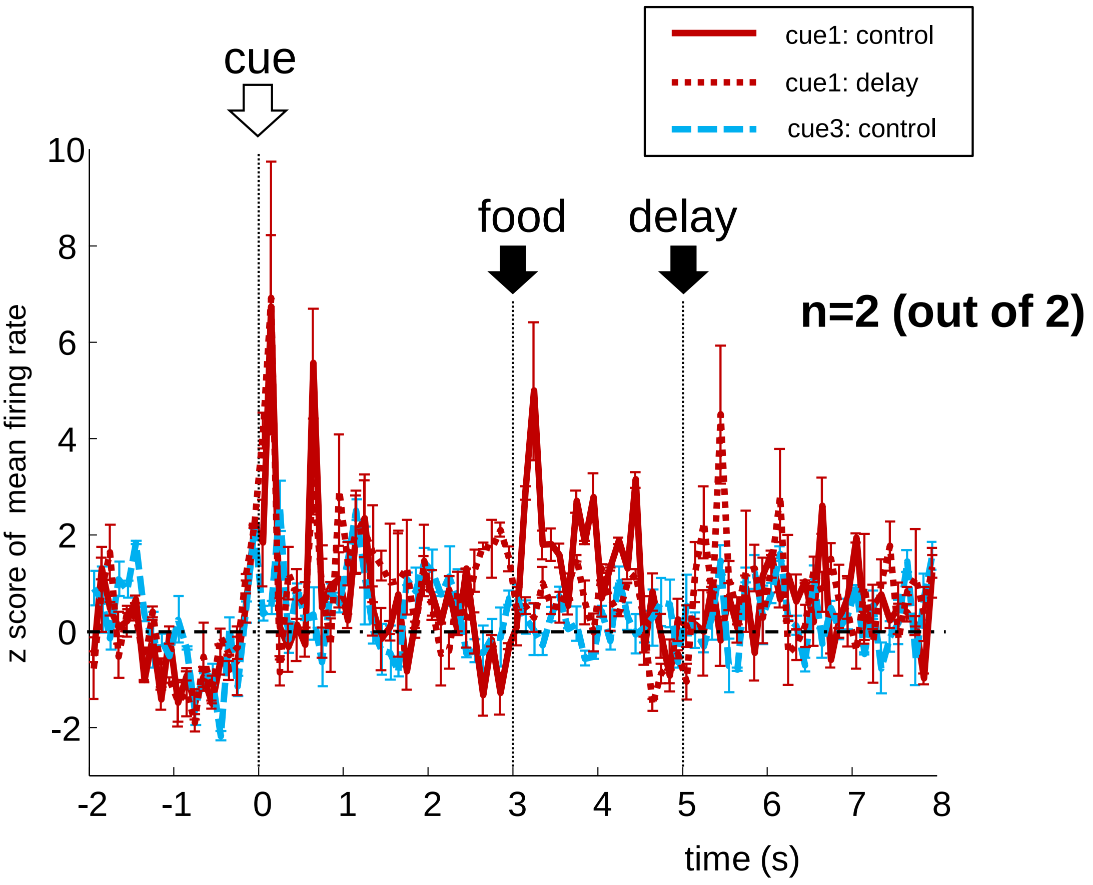

Figure S10. Averaged firing rate of two excitation subtype of the type-3 neurons in tegmentum.

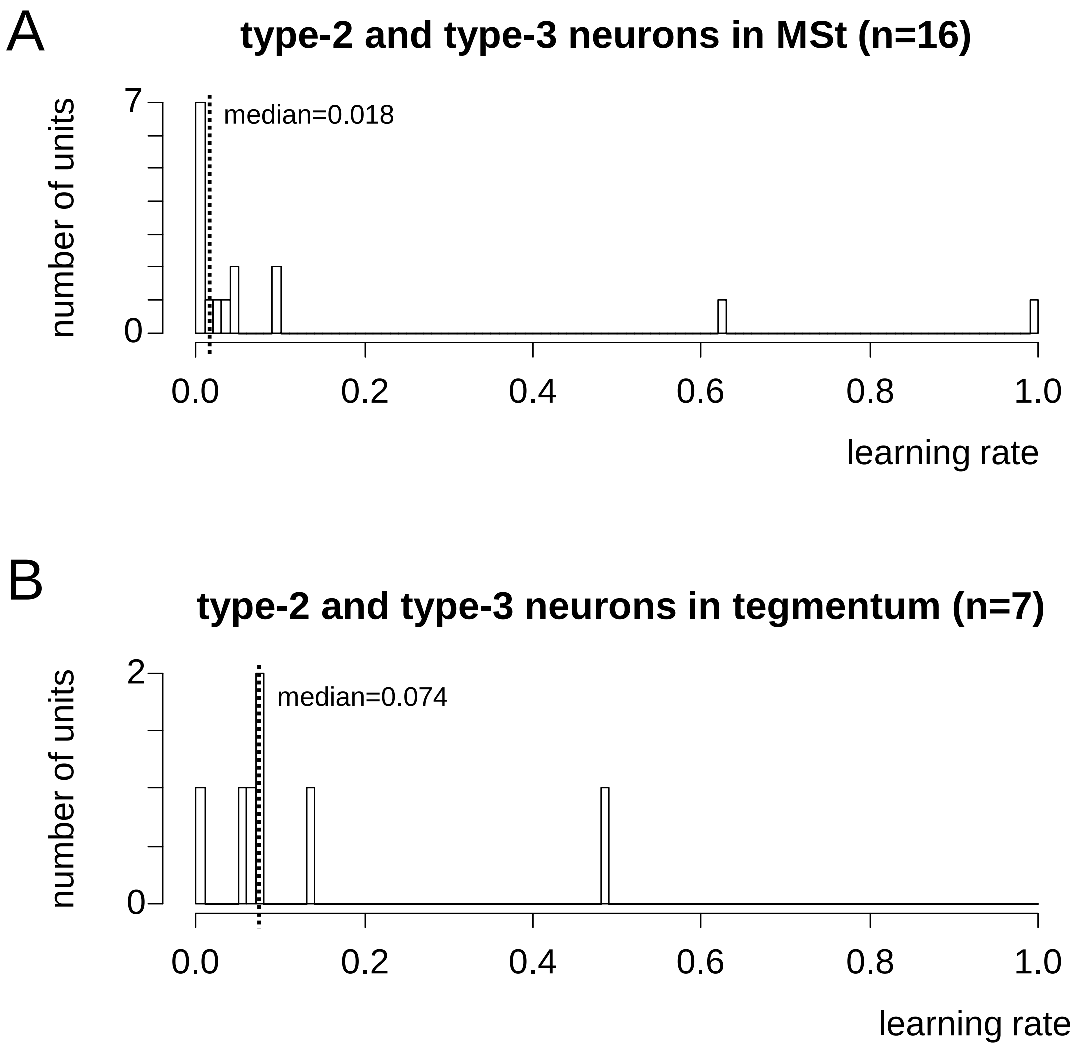

Figure S11. Estimated learning rate (α) of individual neurons. Only the reward-period activities in the type-2 and type-3 neurons are included. (A) MSt. (B) tegmentum.

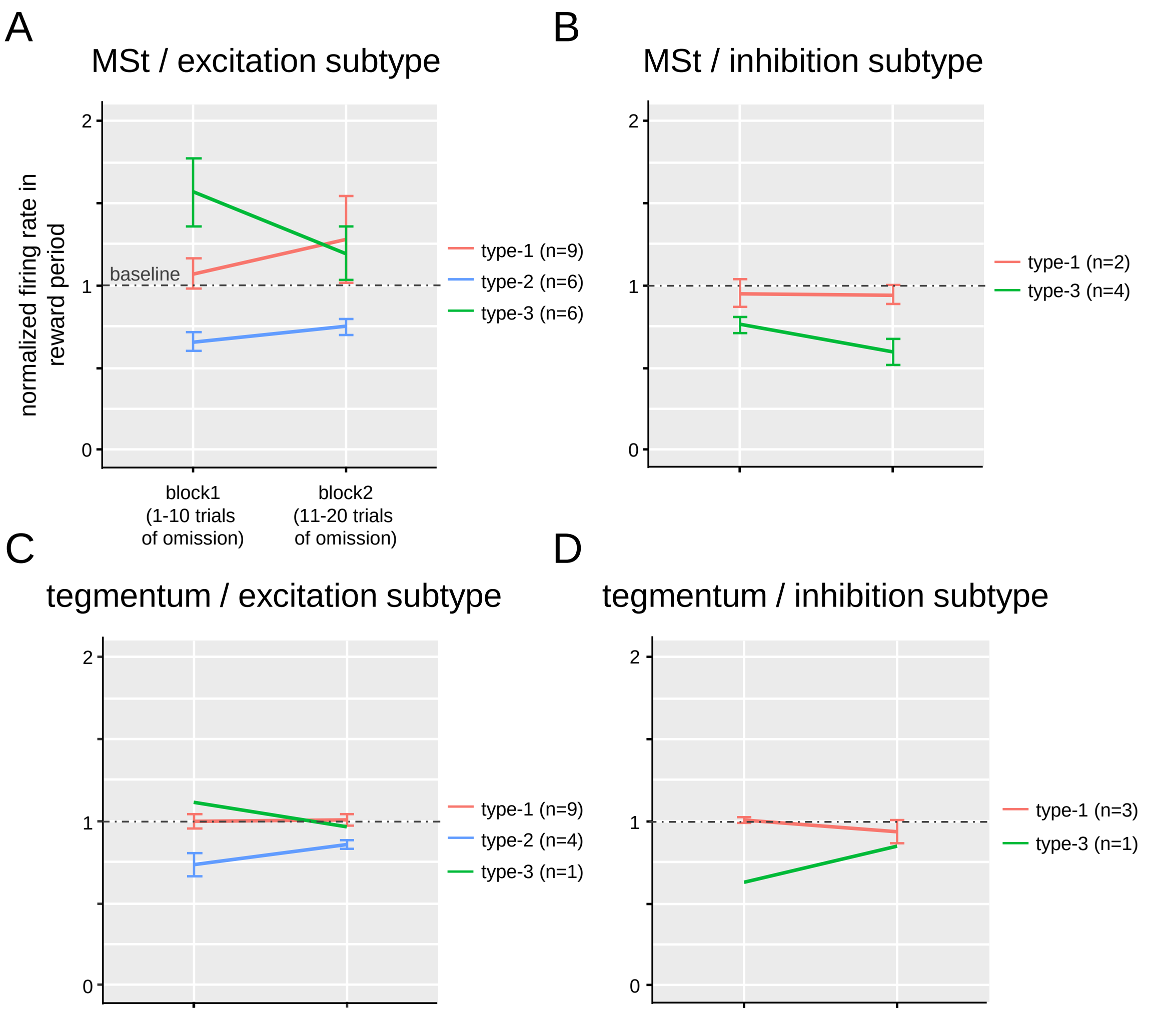

Supplementary figure S12.

Two-way analysis of variance (ANOVA) with repeated measures based on type III sums of squares was applied on the normalized firing rate of categorized neurons. The reward period data of the cue1 trials in the omission block were used. Initial 20 trials of the block were divided into block1 (trial 1-10) and block2 (trial 11-20), each giving rise to an average. Those neurons that were recorded for 20 or more trials in the omission block were included. Groups composed only of one neuron (n=1) were not compared. We used an R function “Anovakun” (developed by Dr. Ryuta Iseki, see below for the URL).

(A) MSt neurons of excitation subtype. Significant main effect occurred in the neuron type ($F_{2,18}$ = 5.6941, p = 0.0121), but not in the block ($F_{1,18}$ = 0.0357, p = 0.8523) or the interaction ($F_{2,18}$ = 1.7274, p = 0.2059). Post-hoc pairwise comparisons after a Holm

correction revealed significant difference between type-1 and type-2 ($df = 18$, $p = 0.0457$), and type-3 and type-2 ($df = 18$, $p = 0.0128$), but not between type-1 and type-3 ($df = 18$, $p = 0.2886$). (B) MSt neurons of inhibition subtype. Significant main effect occurred in the neuron type ($F_{1,4} = 21.7661$, $p = 0.0096$), but not in the block ($F_{1,4} = 0.8978$, $p = 0.3970$) or the interaction ($F_{1,4} = 0.7629$, $p = 0.4317$). (C) Tegmental neurons of excitation subtype. Significant main effect occurred in the neuron type (type-1 and type-2, $F_{1,11} = 14.5217$, $p = 0.0029$), but not in the block ($F_{1,11} = 1.9675$, $p = 0.1883$) or the interaction ($F_{1,11} = 1.3976$, $p = 0.2620$). (D) Tegmental neurons of inhibition subtype. ANOVA was not applicable due to small sample size. Mean ± s.e.m.

In (B) and (C), significant difference appeared among categories of neurons. In (A), on the other hand, no significant interaction occurred between type and block. Probably, inclusion of the type-2 neurons hindered the interaction from being significant. In addition, the type-1 and type-3 MSt neurons (excitation subtype) did not significantly differ in their averaged firing rates. The lack of significant difference is not surprising, because we considered the temporal pattern of changes as critical feature, namely, immediate change occurred after the omission in type-1 neurons, whereas it was gradual in type-3. These feature is detectable by our model selection, but not by ANOVA test of averaged firing rate.

In addition, we must stress that application of ANOVA tests might be inappropriate as it could give a circular double dipping (Kriegeskorte et al. 2009). We categorized neurons by model fitting, and then applied tests, so that the results are inevitably biased to show significant differences. We must be cautious of the ANOVA results.

References:

URL for “Anovakun”: http://riseki.php.xdomain.jp/index.php?ANOVA%E5%90%9B

Kriegeskorte, N., Simmons, W.K., Bellgowan, P.S.F. and Baker, C.I. (2009). Circular analysis in systems neuroscience: the dangers of double dipping. *Nature Neurosci.* 12, 535-540. doi: 10.1038/nn.2303
