# Supplementary material for: Striatal and Tegmental Neurons Code Critical Signals for Temporal-Difference Learning of State Value in Domestic Chicks
Source: Front Neurosci. 2016 Nov 8;10:476. doi: 10.3389/fnins.2016.00476 (PMC5099172; doi:10.3389/fnins.2016.00476)
Supplement: Supplementary file 2 [file DataSheet2.docx]

# Appendix

## Pre-processing of spike data

We performed initial off-line analysis using Spike-2 software (CED Co., Cambridge, UK). To extract single unit activity, a threshold was arbitrarily determined so that the amplitude of the peak (or trough) of spikes exceeded the background noise by 2-fold or more. Spikes were further sorted using a template-matching method and stored as smr files, which were exported to Matlab (version 2009b, The MathWorks Inc., USA) via a library SON provided by CED company.

Spikes were timed in reference to the cue onset as *t* = 0 s in each trial. Spikes were thus counted in each of 100 ms-long bins between *t* = −5 – 15 s, hence there were 200 bins in total for each trial. Trials were sequentially numbered as *T*, with the first trial of the omission block denoted as *T* = 1. We measured firing rate (spikes per *s*) during the reward period (for 2 s or 20 bins during *t* = 3–5 s, if not stated otherwise), denoted as ${FR\_R}_{T}$ for each *T*. The ${FR\_R}_{T}$ was further normalized as a ratio to the corresponding baseline firing rate (*FR_Base* during the period preceding the cue presentation, *t* = −5 – 0 s) as:

${FR\_R\_Nor}_{T}=\frac{{FR\_R}_{T}}{{lowess(FR\_Base)}_{T}}$ (Appendix – 1)

Here, to smooth the fluctuating baseline activity along the procession of trials, we used a built-in R function (lowess) to adopt a robust locally-weighted regression method. In the following, we denote ${FR\_R\_Nor}_{T}$ as *Y_T_* for simplicity. *Y_T_* = 1 means that the reward period was indistinguishable from the baseline inter-trial period in terms of neuronal firing. This normalized reward-period activity *Y_T_* is shown in Figs. 4, 5, 7, 8, e.g. in Fig. 4A(c) and 4C, and in supplementary figures.

## Categorizing neurons based on discrete-time state-space models

We categorized neurons based on their activity during the omission block. To this end, we used the normalized reward-period activities (*Y_T_*) in the cue1 trials. We constructed three types of state-space models; the actual reward model (AR), reward prediction model (RP), and prediction error model (PE). Following the framework employed by Petris et al. (2007), we assumed that each model had one hidden state variable (*θ_T_*) along the discrete trial number, *T*. The firing rate *Y_T_* thus stems from the hidden *θ_T_* in each trial (see each model below). We estimated the parameters of the models using the maximum likelihood method, and chose the best one with the smallest AICs (Akaike Information Criteria) (Akaike, 1974). For model fitting and statistical analysis, we used R (version 3.1.3, R, The R Foundation for Statistical Computing, Vienna, Austria, http://www.r-project.org) in addition to MatLab.

### Actual Reward (AR) model

In this model, we assumed the neurons to be a sensory representation of the actual reward (AR) in each trial, regardless of whether the food was perceived via visual, acoustical, or gustatory clues (i.e. $r_{4}$). The neuronal activity returns to the baseline level as soon as the food is omitted (therefore *θ_T_* = 1 for T ≥ 1), and *Y_T_* is given as:

$Y_{T}=1+\epsilon$ (*T* ≥ 1) (Appendix – 2)

This model therefore does not have a free parameter. Here and the following models, $\epsilon$ is zero-mean Gaussian-distributed noise. The AR model corresponds to the target signal (type-1), specifically in the reward period (Fig. 2C).

### Reward Prediction (RP) model

In this model, we assumed the neurons to represent reward prediction (RP) during the reward period of the trials (i.e. $\hat{V}\left( S_{3} \right)$). Therefore, the hidden variable is *θ_T_* = $p_{T}$ (for T ≥ 1). Following the updating rule (2’) in the results of the main text, the $p_{T}$ asymptotically approaches 1 (meaning no predicted reward) as the omission block proceeds. The observed $Y_{T}$ is thus given as:

$Y_{T}=p_{T}+\epsilon$ (T ≥ 1) (Appendix – 3)

$p_{T}$ is given as follows. The initial value of $p_{T}$ ($p_{0})$ is given by the observed $Y_{T}$ in the last trial of the control rewarding block, after smoothing via the lowess function.

$p_{T}= p_{0}$ (T = 1) (Appendix – 4-1)

$p_{T}$ is subsequently updated at every trial by the product of a learning rate $\alpha\in[0,1]$ and the term representing the prediction error, ($1-p_{T-1}$).

$p_{T}= p_{T-1}+ \alpha\cdot(1-p_{T-1})$ (T ≥ 2) (Appendix – 4-2)

We may reasonably assume that each neuron has a distinct α value as a free parameter. If α is high and close to 1, quick changes will emerge. Alternatively, if α is 0, $p_{T}$ will remain unchanged throughout the omission block. The RP model corresponds to the prediction signal (type-3), specifically in the reward period (Fig. 2C).

### Prediction Error (PE) model

In this model, we assumed the neurons to represent the prediction error (PE) in the reward period (i.e. $\delta_{4}$). Similar to the RP model, we assumed the prediction error ${pe}_{T}$ to be the hidden variable *θ_T_*, and the observed firing rate $Y_{T}$ is given by ${pe}_{T}$ as follows:

$Y_{T}=1+ {pe}_{T}+ \epsilon$ (T ≥ 1) (Appendix – 5)

In the first trial of the omission block (*T* = 1), ${pe}_{T}$ drops considerably ($\Delta R$, reward difference), because the animal does not gain the food that the animal learned to expect during the control block.

${pe}_{T}=\Delta R+(1- \alpha)\cdot{pe}_{0}$ (*T* = 1) (Appendix – 5-1)

In subsequent trials (T ≥ 2), ${pe}_{T}$ will be updated as follows:

${pe}_{T}=(1-\alpha)\cdot{pe}_{T-1}$ (*T* ≥ 2) (Appendix – 5-2)

(5-1) and (5-2) were derived from the updating rule (Appendix – 4-2) in the RP model. See supplementary text S1 for details. The PE model has two free parameters, $\Delta R$ and α. The PE model corresponds to the TD-error signal (type-2), specifically in the reward period (Fig. 2C).

### Fitting neuronal data to the models and selecting the best model

We tested the fit of these three models for the recorded data from each neuron. The free parameters were estimated using the maximum-likelihood estimation method. In the RP model, $\alpha$ was estimated using the built-in function (optimize) in the R programming environment. In the PE model, the two free parameters ($\alpha$ and $\Delta R$) were estimated using the optimx function in the optimx package (Nash, 2014; Nash and Varadhan, 2011). The function (optimx) supplied us with a set of different estimates (14 sets) after applying 14 different optimization algorithms. We chose the one with the highest likelihood among these estimates. To avoid inappropriate estimates, such as local maxima, we graphically checked the fitting curves.

After fitting all three models, we sought to select the best one. We calculated Akaike's information criterion (AIC) for the three models for each neuronal activity data set. The model that yielded the smallest AIC value was thus chosen for the categorization of the neuron under study. To be strict, we added to following criteria post-hoc. Among those neurons classified as AR and RP neurons, the $Y_{T}$ data sets (normalized firing rate during the reward period) were examined for significant activity in the control block. Neurons were ruled out when one-sample *t*-test failed to detect a statistical significance at *p* > 0.05, and the neuron was classified as being an ‘other’ type. The same test allowed us to further classify the neurons (irrespective of AR, RP, and PE type) into two groups: excitation type (positive *t-*value) and inhibition type (negative *t*-value).

## Graphical illustration of neuronal activity

To graphically illustrate the averaged neuron activities, we calculated the z-scores of the spike-counts plotted against the 100 ms bins, averaged across trials. The z-score in the i-th bin is given as:

$z_{i}=\frac{mean\left( C_{i,T} \right)-mean(C\_Base)}{{SD(C\_Base)}/\sqrt{n}}$ (Appendix – 6)

Here, the $C_{i,T}$ is the spike count in trial *T* in the i-th bin. The $C\_Base$ is the set of spike-counts during the period before cue onset (50 bins, *t* = -5 to 0 s). Data obtained for each trial type (e.g. cue1 trials in the control block) were included. We calculated the $mean (C\_Base)$ and the $SD(C\_Base)$ across all bins in the corresponding trial type. SD represents standard deviation, and *n* denotes the number of included trials. The number *n* was fixed constant (n = 20) to reliably compare among different trial types; we excluded neurons if we had recording data for fewer than 20 trials. With this adjusted z-score, positive values indicated the excitatory response and negative values the inhibitory response, in reference to the baseline activity. z-scores above 1.96 or below −1.96 implied a significant excitatory or inhibitory response (*p* < 0.05; no adjustment for multiple comparisons) against the baseline firing rate.

## References

Akaike, H. (1974). A new look at the statistical model identification. *IEEE Trans. Autom. Control* 19, 716–723.

Nash, J. C. (2014). On best practice optimization methods in R. *J. Stat. Softw.* 60, 1–14. Available at: http://www.jstatsoft.org/v60/i02/.

Nash, J. C., and Varadhan, R. (2011). Unifying optimization algorithms to aid software system users: optimx for R. *J. Stat. Softw.* 43, 1–14. Available at: http://www.jstatsoft.org/v43/i09/.

Petris, G., Petrone, S., and Campagnoli, P. (2009). *Dynamic Linear Models with R*. Springer.
